# *In situ* Ti assisted graphitization approach for the preparation of graphite foam with light weight and high thermal conductivity†

**DOI:** 10.1039/d2ra06164c

**Published:** 2023-02-20

**Authors:** Xing Guo, Yaxiong Liu, Xiaodong Tian, Zechao Tao, Xi Yan, Zhanjun Liu

**Affiliations:** a CAS Key Laboratory of Carbon Materials, Institute of Coal Chemistry, Chinese Academy of Sciences Taiyuan 030001 China yanxi@sxicc.ac.cn zjliu03@sxicc.ac.cn; b Center of Materials Science and Optoelectronics Engineering, University of Chinese Academy of Sciences Beijing 100049 China; c Dalian National Laboratory for Clean Energy Dalian 116023 China

## Abstract

The state-of-the-art graphite foams (GFs) are afflicted by large bulk density and low thermal conductivity, restricting their practical application. To alleviate the above problem, herein, an issue-oriented scheme, *i.e.*, an *in situ* titanium (Ti) assisted catalytic graphitization strategy was proposed by using AR mesophase pitch (ARMP) as a precursor. In a typical preparation process, the mixture of Ti and ARMP underwent a pressurized foam, carbonization, and graphitization procedure successively to obtain GFs. The results showed that the Ti content played an important role in the development of the graphitic microcrystal structure due to the catalytic graphitization of Ti. According to the XRD analysis and molecular dynamics (MD) simulation, we confirmed that Ti promoted graphitization mainly by the generation of TiC during the high-temperature graphitization. The GFs obtained with 11 wt% Ti exhibited the most perfect graphitic crystal structure, with the highest graphitization degree. Thanks to the improved graphitization degree, the thermal conductivity of GFs increased with the added amount of Ti increasing from 0 to 11 wt%. The highest thermal conductivity of 60.8 W m^−1^ K^−1^ and the low bulk density of 0.36 g cm^−3^ could be achieved when the addition amount of Ti was 11 wt%. Meanwhile, apart from the optimization of thermal conductivity and bulk density, the compressive strength was also enhanced as the amount of Ti increased from 0 to 15 wt%. Our work provided a facile and scalable approach to preparing GFs with low density and high thermal conductivity.

## Abstract 2

In this study, a lightweight and high thermal conductivity graphite foam with perfect crystal structure and superior graphitization degree was fabricated *via in situ* titanium assisted catalytic graphitization strategy.

## Graphical abstract

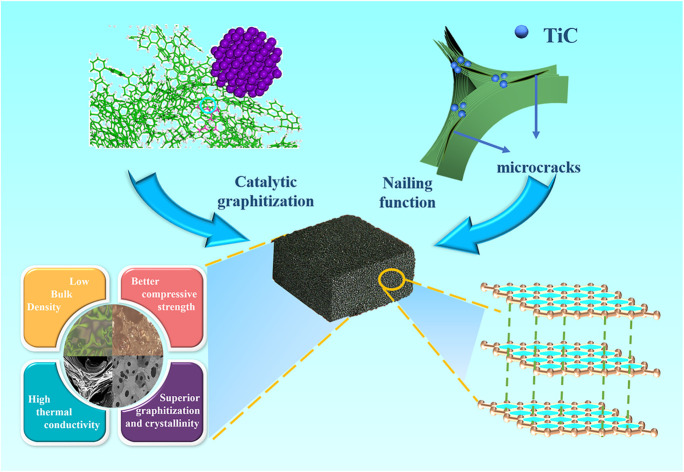

## 1. Introduction

Owing to the combining merits of tailor-made thermal conductivity, open-cell structure, bulk density and thermal expansion coefficient, graphite foams (GFs) have triggered broad attention in thermal management systems for aircraft, energy storage power stations, and consumer electronics^1–6^. In addition, the rich internal pore structure and conductive properties of GFs also make them promising in the field of electromagnetic interference shielding with the burgeoning development of electronic devices recently.^7,8^ From the perspective of lightweight portable electronic device application,^9,10^ it is urgently desirable for cutting-edge heat conductive materials with superior thermal conductivity and low bulk density.

Up to now, various GFs have been prepared and their thermal properties depend on the used precursor to a large extent.^11^ For example, GFs with high compressive strength of 11.72 MPa were prepared by using coal-tar pitch as a precursor. Unfortunately, the thermal conductivity of as-obtained GFs was less than 22 W m^−1^ K^−1^ and the density was up to 0.70 g cm^−3^, which is far from meeting the practical usage criteria.^12^ In this line, tremendous endeavors have been made to exploit lightweight GFs with high thermal conductivity during the past decade. In Abhay's work, polyurethane foam was adopted as a template to prepare GF, the as-prepared GF exhibits a high thermal conductivity of 60 W m^−1^ K^−1^ at a bulk density of 0.58 g cm^−3^ (ref. 13). The bulk density of GF prepared by Walter *et al.* could be achieved as low as 0.25 g cm^−3^ by combining the coal tar mesophase pitch and expanded graphite. However, the thermal conductivity was still unsatisfactory (21 W m^−1^ K^−1^).^14^ By regulating the relative content of ultrafine graphite in AR mesophase pitch (ARMP), the highest thermal conductivity of 43.2 W m^−1^ K^−1^ was achieved at a bulk density of 0.45 g cm^−3^.^15^ According to the literature report that the ARMP molecules are more like a rod in nature and the typical coal tar mesophase pitch is more like a disc.^16^ This rod-like molecular structure has a well-orientation and facilitates the extension of the graphite structure at the ligaments.^17^ Combined with the above works, we can find that ARMP is acclaimed as an ideal precursor for the preparation of GFs with high thermal conductivity because ARMP is easier to form a well-developed graphite microcrystal structure during the heat treatment process, which is conducive to the improvement of thermal conductivity. A delicate trade-off between thermal conductivity and bulk density was gained to some extent, it is still far from actual demands.

As we all know, thermal conductivity is related to the graphitization degree.^18^ Generally, the carbon materials with higher graphitization degree process larger graphite crystallite size, resulting in better thermal performance. Therefore, improving the graphitization degree of GFs is expected to augment their thermal conductivity. Catalytic graphitization is perceived as an effective method to enhance the graphitization degree of carbon materials.^19^ The catalyst plays a key role in the reconstruction of the structure, transforming the matrix from a disordered carbon layer structure to a perfect graphite microcrystalline structure.^20^ Nowadays, numerous catalysts such as elementary substances (Fe, Co, Ni, Ti, V, Cr, Mn, and B),^21–23^ metal oxides (Fe_2_O_3_,^24,25^ Cr_2_O_3_, and MnO_2_ (ref. 26)) and alloys (Fe–Si alloy^27^) have been investigated based on dissolution/reprecipitation and formation/decomposition mechanism of metal carbides.^28–31^ Among them, Fe and Cr can improve the degree of graphitization of the C/C composites at the cost of the deterioration of strength to some extent.^32^ While Mn and B can only link the disordered lamellar structure into sheets and is unable to directly transform the amorphous carbon into an ideal graphite structure.^33^ In contrast, the Ti element could boost the graphitization degree based on the formation/decomposition mechanism of TiC during high-temperature graphitization. It is worth noting that the presence of TiC is inclined to improve the strength of materials.^34,35^

Molecular dynamics (MD) simulation is the closest simulation method to experimental conditions in molecular simulation, which can give the microscopic evolution of the system from the atomic level and visualize the mechanism and law of the occurrence of experimental phenomena. Thus, MD simulation is prompting our research to develop in a more efficient, economical, and predictable direction.^36^ Tang *et al.* investigated the catalytic conversion process of Fe_2_O_3_ from a molecular perspective and the results demonstrated that Fe_2_O_3_ is reduced to Fe at high temperatures.^37^ Their work paves the way for exploring the mechanism of catalytic graphitization at the molecular level using MD.

Inspired by this, the *in situ* Ti assisted catalytic graphitization strategy was chosen to prepare high-performance GFs by using ARMP as the carbon precursor in this work. The effects of Ti content on the evolution of microstructure were systematically investigated. MD simulation was also conducted to gain a deep insight into the catalytic process. Thanks to the improved graphitization degree, the thermal conductivity of GFs increased with the added content of Ti increasing from 0 to 11 wt%. The highest thermal conductivity of 60.8 W m^−1^ K^−1^ and the low bulk density of 0.36 g cm^−3^ could be achieved when the addition content of Ti was 11 wt%. Meanwhile, apart from the optimizing of thermal conductivity and bulk density, the compressive strength was also enhanced up to 68% as the content of Ti increased from 0 to 15 wt%.

## 2. Experimental section

### 2.1 Materials

In this research, Mitsubishi ARMP was selected as the precursor. ARMP was pulverized into granules with an average size of 150 μm for further utilization. The properties of ARMP were listed in Table S2.† The Ti powders (99.9 wt%, 325 mesh, Aladdin) were further ground. The polyoxyethylene was purchased from Shanghai Chemical Dispensing Factory (99.9 wt%) and used without further purification.

### 2.2 Preparation of Ti-doped GFs

**Fig. 1:**
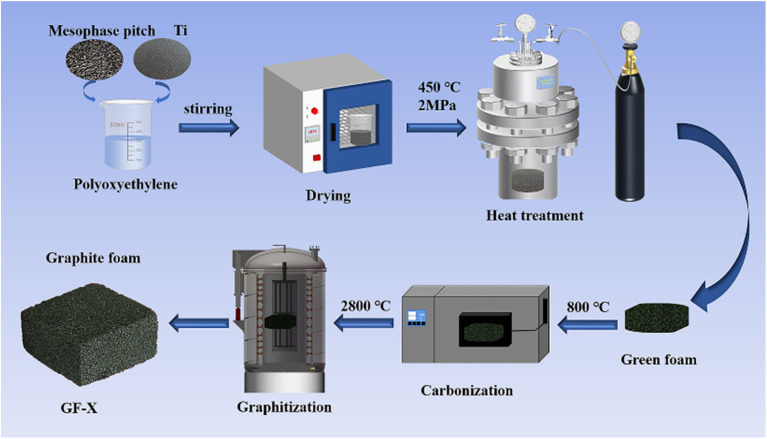
Schematic diagram of the fabrication of Ti-doped graphite foam.

Fig. 1 illustrated in the schematic diagram of Ti-doped GFs. ARMP and Ti dopant were first mixed in the polyoxyethylene solution. Then, the mixture was dried at 150 °C for 8 h. After drying, the Ti-containing mixture was heated up to 450 °C under 2.0 MPa with a heating rate of 1 °C min^−1^ in an autoclave and dwelled for 2 h at the final temperature to get green foams. Subsequently, the carbonization of obtained green foams was performed at 800 °C in Ar atmosphere with a heating rate of 2 °C min^−1^ and maintained at the final temperature for 2 h. Finally, the carbonized foams were graphitized to 2800 °C in the Ar atmosphere and dwelled for 1 hour to obtain GFs. The prepared GFs were denoted as GF-*X*, where *X* referred to the Ti content in ARMP (*X* = 0, 3, 5, 7, 11, 15 wt%).

### 2.3 Characterizations

The ARMP was encapsulated in the mold with epoxy resin, polished, and ground using different grit sandpaper and alumina powder. A surface image of the ARMP was acquired on a Leica DM2700P using polarizing microscopy (PMO) technology with a 20× objective. It could be used to observe and analyze the optical texture and mesophase content. The surface chemical properties of the ARMP were researched by Fourier Transform Infrared Spectroscopy (FTIR, VERTEX 80v, Germany). The ARMP was mixed with KBr for tableting at a ratio of 1 to 200, with a scanning range of 500–4000 cm^−1^. The contents of the ARMP were detected by an elemental analyzer (EA, Thermo Scientific, USA). The gases escaping from the ARMP were measured using TG-MS (TG, STA 409PC, Germany, and MS, QMS 403C, Germany) to explore the foaming process at a ramping rate of 5 °C min^−1^ in the Ar atmosphere. A viscometer (KVDV-3) was used to record the viscosities of the mixture of ARMP and Ti dopant as a function of temperature. A scanning electron microscope (SEM, Japan, JSM-7100) was used to observe the microstructure of GFs. The distribution and chemical composition of dopant particles were characterized by backscattered-electron (BE) and the X-ray energy dispersion spectrometer (EDS). The pore structure and pore size distribution inside the GFs were observed by industrial computed tomography (CT, YXLON-FF35, Germany). The microcrystalline structure of GFs was obtained by X-ray diffractometer (XRD, Bruker D8, Germany), using Cu Kα radiation (*λ* = 0.15418 nm), transmission electron microscopy scanning (TEM, JEM-2100F, Japan) and selected area electron diffraction (SAED). The crystalline parameters were obtained using the Bragg eqn (1) and Scherrer eqn (2).1
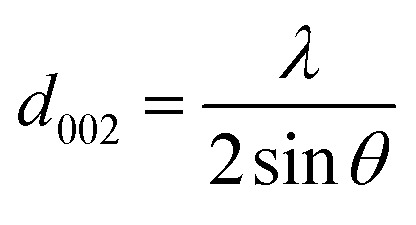
2
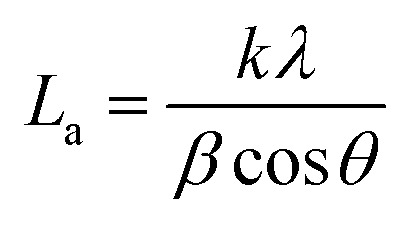
where *L*_a_ is the average crystallite size in the sample, *λ* is the X-ray wavelength (0.15406 nm), *d*_002_ is the graphite interlayer spacing, *β* and *θ* are the full-width half maximum of the diffraction peak and Bragg diffractive angle respectively.^38^ The degree of graphitization (*G*) was calculated by the Mering–Maire formula.^39^3
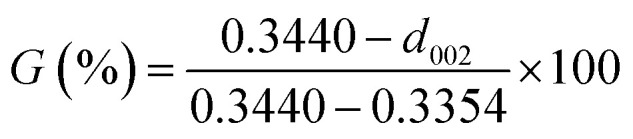
where *d* is the average crystalline spacing of the test sample (002); 0.3440 is the crystalline spacing (nm) of the completely disordered structure (002); 0.3354 is the crystalline spacing (nm) of the ideal graphite (002).

The lattice defects of GFs were investigated on a Lab RAM HR Evolution Raman Spectrometer (Horiba, UK) with an excitation wavelength of 532 nm radiation. The degree of graphitization of carbon materials refers to the degree of ordering of the material inversely proportional to *R* (*R* = *I*_D_/*I*_G_). A larger *R*-value indicates more defects in graphite crystals, while a smaller *R*-value means higher degree of graphitization and closer to the microcrystalline structure of graphite.^40^

The compressive strength of GFs was measured by an Instron 5592 universal testing machine. The GFs were first cut into blocks of 10 × 10 × 3 mm^3^, and the thermal diffusivity (*α*) of the GFs was then measured on the Netzsch LFA447/2-2 InSb nano flash machine at 25 °C. The *C*_p_ of all GFs was obtained by the Differential Scanning Calorimetry (DSC) method on the DSC200 F3 MAIA at 25 °C. The thermal diffusivity (*α*), density (*ρ*), and *C*_p_ (Table S1†) of GFs were used to calculate the thermal conductivity (*κ*) with the following relation:

4 *κ* = *α* × *ρ* × *C*_p_

The catalytic graphitization process of ARMP molecules over Ti particles was investigated using ReaxFF MD simulation.^17,41–43^ ReaxFF forcefield used a general relationship between bond distance and bond order and between bond order and bond energy, leading to proper dissociation of bonds. 20 ARMP molecules and a cluster of 120 Ti atoms were put in a 50.0 × 50.0 × 80.0 Å^3^ (*X* × *Y* × *Z*) periodic box to generate a model system (Fig. S1†). The relevant parameters were displayed in the ESI.† The same ratio of Ti components as in the experiment (7 wt%). To investigate the catalytic graphitization behavior, the model system was set at 2727 °C for 600 ps. During the ReaxFF MD simulation, the atomic coordinates were saved every 1 ps for post-analysis.

## 3. Results and discussion

### 3.1 Structures and properties of ARMP

**Fig. 2:**
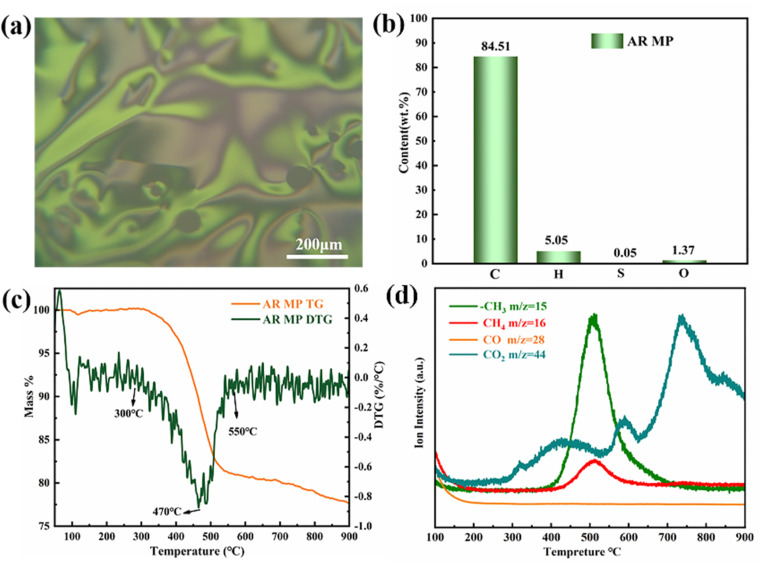
(a) Polarizing microscope images of ARMP. (b) The element content of ARMP. (c) TG-DTG curve and (d) mass spectra of ARMP.

As presented in Fig. 2a and Table S2,† the ARMP showed high carbon content of 84.51 wt% with a full percent of mesophase, making it more suitable for the preparation of GFs with well-ordered microcrystalline structures. These basic features were apt to foster thermal conductivity. When taking the low ash content and high volatile characteristics into consideration, ARMP is more of a concern than coal tar pitch. Because ARMP has the potential to fabricate GFs with low bulk density and optimized porous structure.^44^ According to Fig. 2a, anisotropic structures with flow domain optical texture (>60 μm long, ≥30 μm wide) could be found under polarized light microscopy.^45^ The multiple flow domain structure is propitious to the orderly arrangement of the carbon layer structure within the GFs.^46^ These features imply that this ARMP will favor the formation of GFs with high thermal conductivity. Fig. S2† presented the FT-IR spectra of the ARMP, showing typical fused ring aromatic structures. Three peaks occur were found at 1600 cm^−1^, 2915 cm^−1^, and 3040 cm^−1^, which were attributed to the C=C bond stretching vibration peaks of aromatics, the C–H bond stretching vibration peaks corresponding to aliphatic hydrocarbons and aromatics, respectively. However, 1440 cm^−1^ was the C–H bond bending vibration peak of methylene.^47^

In order to probe the foaming process, the TG-DTG curve of the ARMP was remarked at the temperature range of 46–900 °C in the Ar atmosphere. As presented in Fig. 2c, evidenced weight loss peak at 300–550 °C could be observed corresponding to the decomposition of aliphatic side chains and methyl side chains on the aromatic ring in the ARMP.^48^ They mostly evaporated in the form of light fractions. Multi-weight loss peaks meant the light fractions in coal tar pitch with multistage distributions.^49^ However, the single-peak weight loss indicated the narrow range distribution of light fractions in the ARMP, benefiting the control of the foaming process. From the Mass curves (Fig. 2d), the release of –CH_3_ and CH_4_, which was associated with the demethylation process, increased significantly from 400 °C to 773 °C, implying the cracking of side chains molecules on the aromatic ring in the ARMP. The release of CH_4_ and –CH_3_ from ARMP left active sites on the aromatic ring, where the aromatic ring molecules were cross-linked by condensation.^50,51^ Inevitably, CO_2_ and other carbon oxides were released by the decomposition in the whole process. The escape of gases during the pyrolysis of ARMP eventually affected the pore structures and distribution of GFs. These processes determine the pore-wall size and the density of the GFs.

### 3.2 Effect of Ti-doped content on the pore structure of GFs

**Fig. 3:**
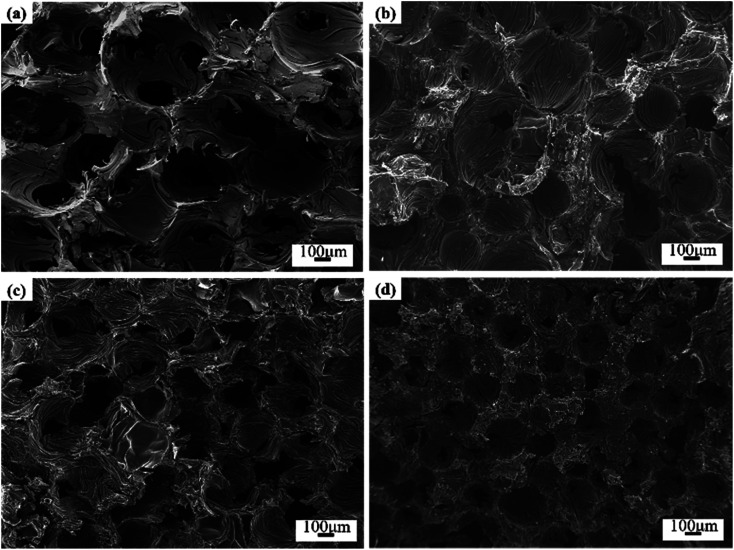
SEM micrographs of Ti-doped GFs (a) GF-0, (b) GF-7, (c) GF-11 and (d) GF-15.

Fig. 3 showed the SEM micrographs of Ti-doped GFs. The image of GF-11 taken from the camera could be referred to in Fig. S3.† The open-cell structure with different pore sizes and wall thicknesses could be seen in all GFs. The open-cell diameter of GFs decreased with the increase of Ti content, while the cell wall thickness increased accordingly. Thus, the addition of Ti influenced the cell morphology in aspects of open-cell diameter and cell-wall thickness, which might be caused by the variation of viscosity.^11^

**Fig. 4:**
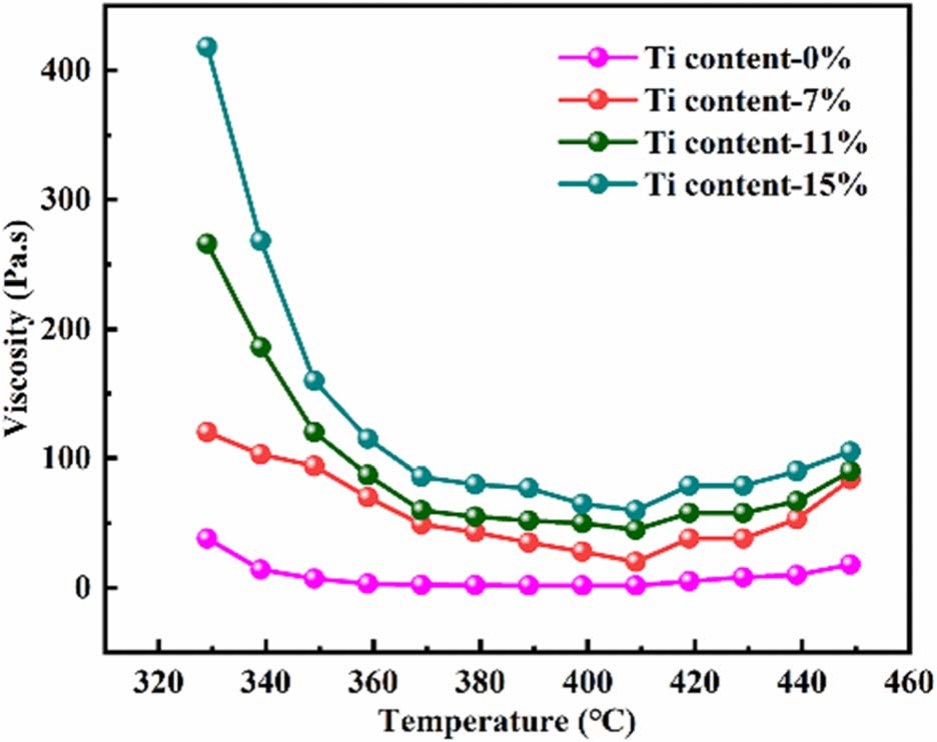
Viscosity of pure ARMP and the mixture of ARMP with different content of Ti at different temperatures.

The viscosity of the mixture is important for the foaming process, which greatly relies on the temperature. Therefore, the viscosity of the hybrid of ARMP and Ti as a function of temperature was discussed and the results were presented in Fig. 4. As shown in this figure, all mixture exhibited a similar tendency, namely that the viscosity decreased first and then increased slightly as the temperature increases. To be specific, the viscosity of the mixture increased obviously along with the addition of Ti. With increasing temperature, the viscosity decreased in all the systems, minimizing it at 407 °C, which was the result of the stronger fluidity after the ARMP melted. As the temperature continued to rise, the presence of Ti as the nucleation site could promote the evaporation of low-molecular-weight units in the ARMP, which caused ARMP to condense into macromolecules, resulting in an increase in viscosity.^52^ Variations in viscosity might contribute to differences in the behavior of stretching during the bubble formation and growth phases, and consequently affect the evolution of cell structures.^53,54^ When the Ti content was low, the mixture showed poor elasticity and facilitated bubbles development, leading to enlarged open-cell sizes in the low viscosity. On the contrary, high viscosity could hinder the evolution of pyrolysis gases making it difficult to bind the larger bubbles, resulting in smaller open-cell sizes.

**Fig. 5:**
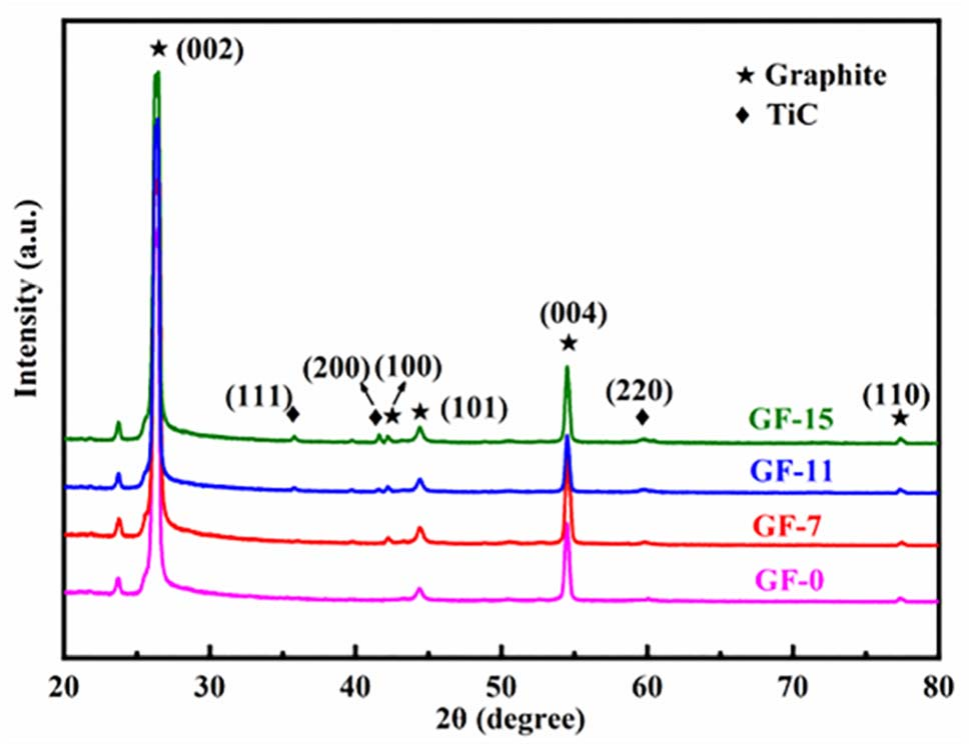
X-ray diffraction patterns of GFs with different amounts of Ti.

XRD patterns were recorded to analyze the effect of Ti doped on the graphitization process. As displayed in Fig. 5, apart from the characteristic peaks of the graphite, TiC was detected in Ti-involved GFs, indicating that Ti was transformed into TiC during graphitization. Additionally, the GFs doped Ti exhibited narrow and asymmetric (002) diffraction peaks, indicating the presence of a highly ordered graphitic structure. Besides, the (002) peak shifted to higher degree after the addition of Ti, which suggested an improved graphitization degree. The calculated *d*_002_ and *L*_a_ listed in Table 1 also verified the positive effect of Ti on graphitization. The graphitization degree reached the highest level of 94.1% with an interlayer spacing of 0.3359 nm when the Ti doped was 11 wt%. The value of *L*_a_ increased from 86.4 nm of GF-0 to 190.0 nm of GF-11. Since the crystalline size of the carbon material determined the upper limit of the thermal conductivity.^55^ GF-11 with the largest crystallite size will show satisfied thermal conductivity compared with other GFs prepared in this work.

**Table 1:** Crystal parameters and degree of graphitization of GFs

Samples	GF-0	GF-3	GF-5	GF-7	GF-11	GF-15
Ti content (wt%)	0	3	5	7	11	15
2*θ* (degree)	26.47	26.47	26.48	26.50	26.52	26.51
*d*_002_ (nm)	0.3365	0.3364	0.3361	0.3361	0.3359	0.3360
*L*_a_ (nm)	86.4	95.0	135.7	135.7	190.0	176.0
*G* (%)	87.2	88.0	92.3	92.3	94.1	93.2

**Fig. 6:**
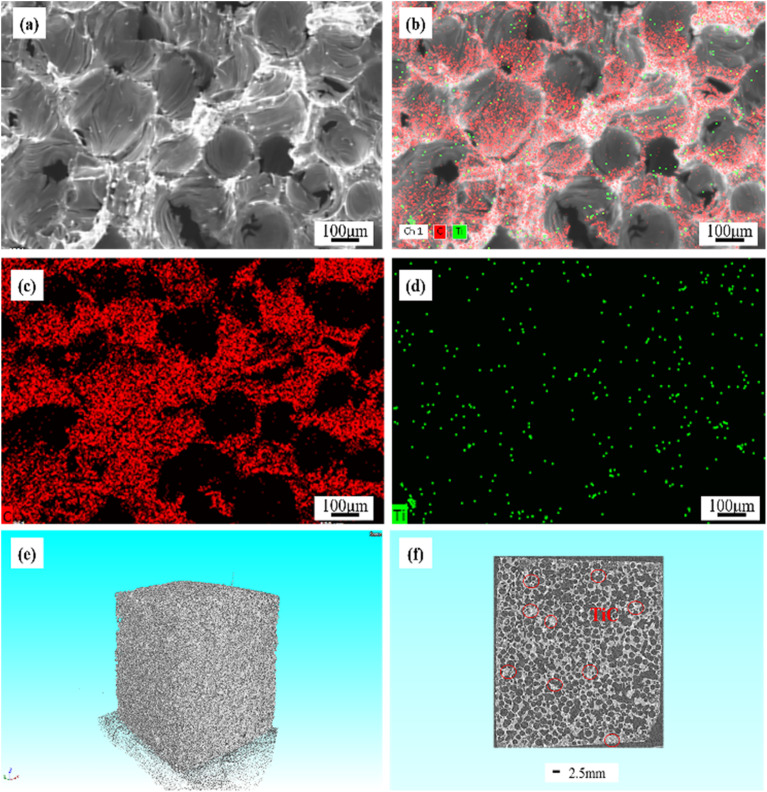
EDS mapping analysis (a)–(d) of GF-11, high-resolution CT images of GF-11 (e and f).

Fig. 6b–d showed the EDS mapping analysis of GF-11. The obvious distribution of Ti and C elements in the graphite foam was observed. Based on XRD results in Fig. 5, it was known that Ti finally existed in the form of TiC. As depicted in Fig. 6b and d, TiC particles were uniformly distributed in GF-11 and no impurity could be detected. The overall morphology and the internal pore structures of the GF-11 were collected by tomographic section scanning of the high-resolution CT. Ample pores were evidenced from the black and white stereo picture shown in Fig. 6e. The CT scanning image presented in Fig. 6f, from which one could figure out that TiC was uniformly distributed on the pore walls, which played an important role in strength enhancement for GFs. The even-distributed TiC particles in the GFs made the overall plastic zone more homogeneous. In this architecture, the internal stress could be evenly distributed. Thus, no local strain concentrations were generated when the load was applied, causing small deformations in the internal structure of the GFs.^56^

**Fig. 7:**
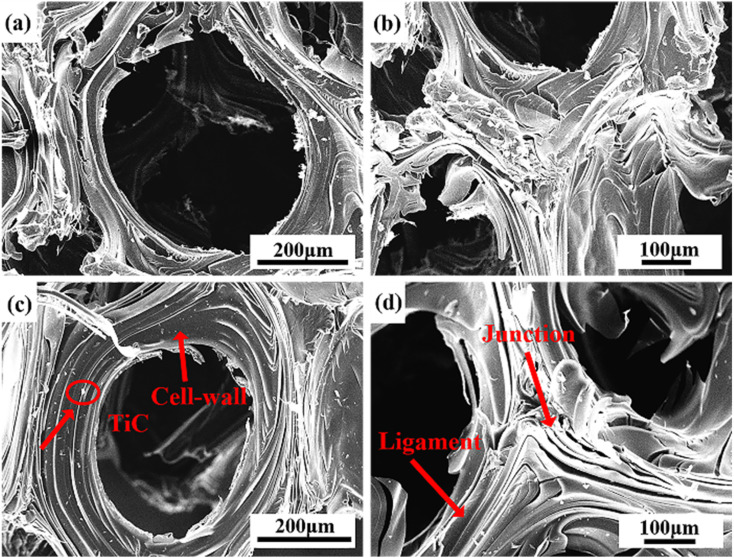
SEM images of GF-0 (a and b) and GF-11 (c and d).

Fig. 7 displayed the SEM images of the cell wall and ligament of GF-0 (a and b) and GF-11 (c and d). In contrast, GF-11 exhibited a better-aligned graphite laminar ligament structure. The orderly-arranged ligament structure paved the way for the elevation of thermal conductivity.

**Fig. 8:**
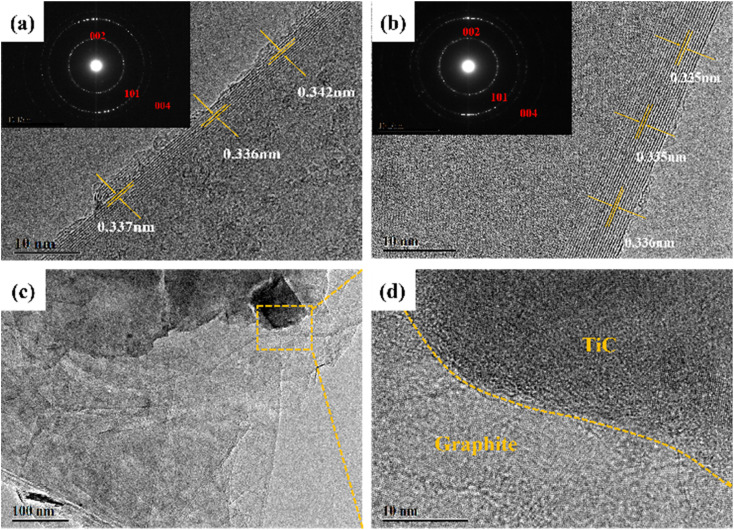
TEM images of GF-0 (a) and GF-11 (b–d).

In order to detect the microcrystalline structure, the HRTEM images were also illustrated. The SAED patterns in the inset of Fig. 8a and b exhibited the (002), (101), and (004) planes, authorizing the existence of graphite. Compared with the image shown in Fig. 8a, the GF-11 displayed more obvious graphitic stripes in Fig. 8b. It is verified that Ti improved the graphitization degree of GF, which further demonstrated that Ti could promote the graphitization process. The GF-11 graphite flakes were stacked in an ordered manner in Fig. 8c, which suggested a high degree of graphitization. TiC and graphite developed complex interfacial structures inside the GFs during the graphitization process. In Fig. S4,† the obvious interface between the edge of the TiC particles and the graphite layer was observed in the GF-11, where Ti and C showed a uniform distribution. Thus, in Fig. 8d, it could be seen that the transition between the interfaces of TiC and graphite was natural and well-integrated, which was very meaningful for regulating the stress distribution and effective load transfer within the GFs.^28^

**Fig. 9:**
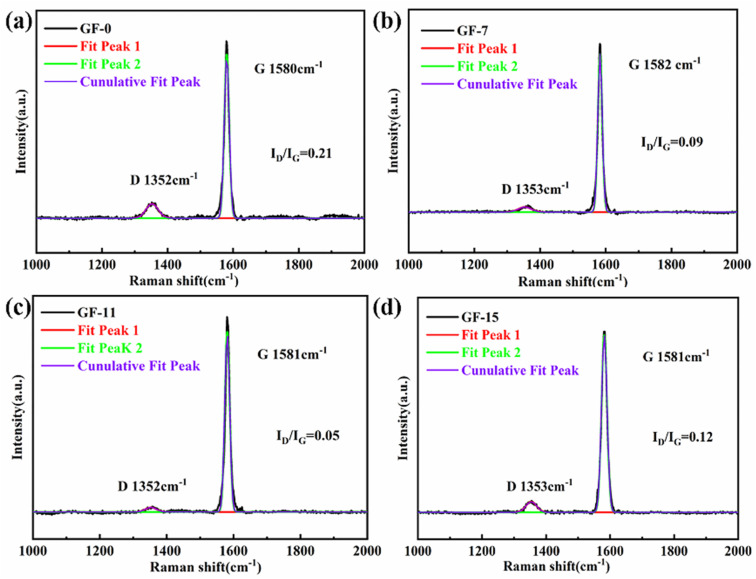
Raman spectra of GF-0 (a), GF-7 (b), GF-11 (c), GF-15 (d).

The graphitization degree of the GFs was sensitive to the content of the Ti dopant. Raman spectra in Fig. 9 evidently demonstrated this phenomenon. It was well accepted that the intensity ratio (*R*) of the D peak and G peak, located around 1350 and 1580 cm^−1^. It could be used as an index to estimate the graphitization degree of the obtained carbonaceous materials. This paper focused on the G and D peaks, neglecting other features that are sometimes present (bands D′ or D2 and D3). When the content of Ti increased from 0% to 11 wt%, the *R*-value decreased from 0.21 to 0.05. The decrease is as much as around 4 times. The decrease in *R*-value indicated a substantial reduction of microcrystalline defects inside the GFs, meaning that the Ti content positively affected graphitization. Ti acted as a catalyst for graphitization promoting the formation of internal graphite microcrystals. The GFs inside tended to become an ideal graphite microcrystalline structure with a high degree of orientation, which played a significant role in the enhancement of thermal conductivity. When the Ti content increased from 11% to 15 wt%, the *R*-value sharply improved from 0.05 to 0.12, which indicated that the defects in the graphite lattice were dramatically increased. This was probably the formation of a large amount of TiC at high temperatures, which introduced an excess of defects.

### 3.3 Effect of Ti-doped content on the performance of GFs

**Fig. 10:**
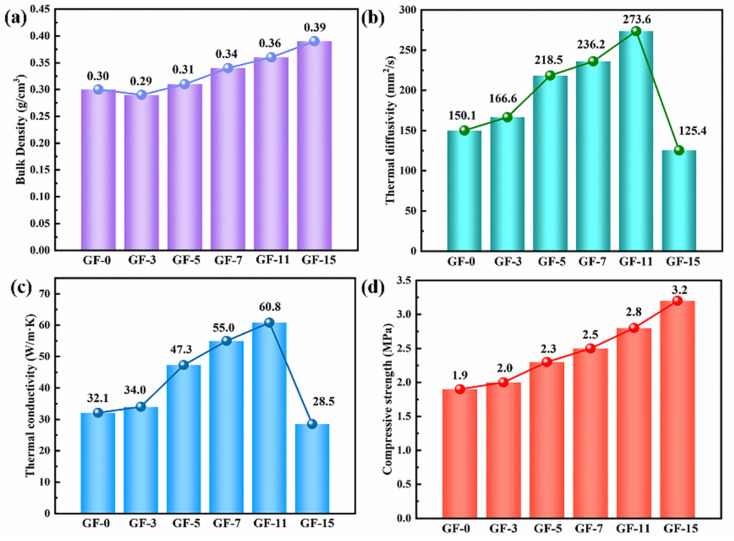
The bulk density (a), thermal diffusivity (b), thermal conductivity (c) and compressive strength (d) of as-obtained GFs.

Fig. 10 showed the bulk density, thermal diffusivity, thermal conductivity, and compressive strength of GFs as a function of Ti content. The bulk density of GFs increased from 0.30 to 0.36 g cm^−3^ due to the generation of TiC (Fig. 10a). The thermal diffusivity showed the same trend as thermal conductivity (Fig. 10b). It could be noticed that the thermal conductivity of GFs without Ti was only 32.1 W m^−1^ K^−1^, and it reached a maximum value of 60.8 W m^−1^ K^−1^ when 11 wt% Ti was involved (Fig. 10c). The delicate balance of high thermal conductivity and relatively low density made the GF-11 promising in light-weighted thermal conductive applications. Meanwhile, our work also affirmed the exceptional advantage of Ti doped for the fabrication of lightweight GFs doped Ti with high thermal conductivity compared with previously reported ultrafine graphite, zirconium, graphitized carbon black, carbon fiber, MCMBs, *etc.* (Table 2).

**Table 2:** Properties of GFs prepared with different additives

Samples	Additives	Thermal conductivity (W m^−1^ K^−1^)	Density (g cm^−3^)	Ref.
GF20	Ultrafine graphite	42.80	0.49	15
CF3	Zirconium	63.00	0.72	57
GF_0_	Carbon black	39.10	0.76	58
CF6	Carbon fiber	83.00	0.80	59
CF1–55	MCMBs	43.70	0.78	60
GF11	Ti	60.80	0.36	This work

Of course, the thermal conductivity of GFs decreased when Ti content was continually increased. The disorder of the composite system increased with the generation of large amounts of TiC, which led to a decrease in the crystallite size in the horizontal direction (*L*_a_) and an increase in defects. This was also confirmed by the study of Raman spectroscopy (Fig. 9). A vast number of phonons were scattered at the defects, resulting in ineffective heat transfer. Therefore, the introduction of excessive Ti was not favorable to the thermal conductivity of the GFs.^29^

Except for bulk density and thermal performance, Ti doped also affected the compressive strength of GFs. As depicted in Fig. 10d, the compressive strength increased by 68% after 15 wt% Ti was introduced. According to the brittle materials fracture rule, the mechanical strength of carbon materials was related to the length and quantity of cracks in blocks.^61^ The possible reason for the appearance of microcracks at cell walls and junctions was the mismatch of coefficient thermal expansion between in-plane and out-of-plane graphitic layers developed from large anisotropic flow domains.^53^ Therefore, the lesser the microcrack in GFs, the better the mechanical strength the GFs had. Ti-doped GFs had fewer microcracks than that pure GF, so they possessed better compressive strength (Fig. 7).

**Fig. 11:**
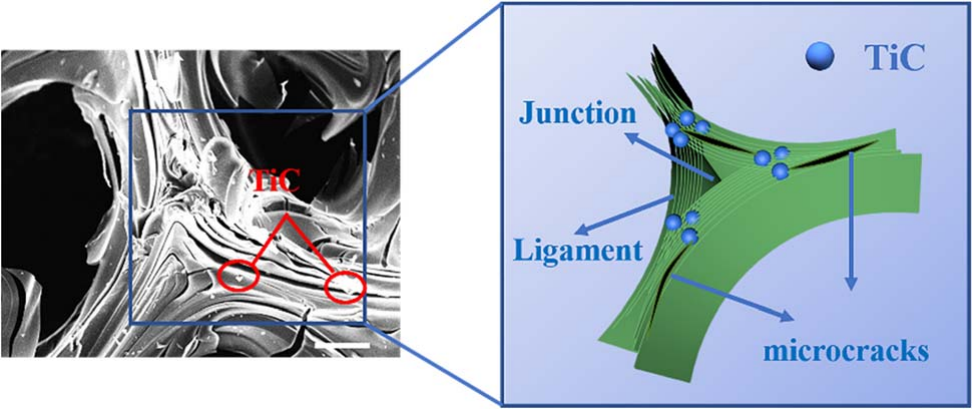
Schematic diagram of structural enhancement of GF-11.

The well-dispersed TiC particles in the GFs could evenly absorb the thermal stresses generated during graphitization, thus serving to “nails” the cracks. “Nails” not only effectively inhibited the cracking of the laminar structure on the pore wall but also effectively prevented the cracking at the edge of the ligament and the junction region in the GFs. As illustrated in the schematic (Fig. 11), we supposed that the TiC particles act like nails to hold the microcracks together.^62^ When the length of the microcrack was enlarged, the TiC particles in the GFs would prevent microcracks from growing up. In addition, the TiC particles would deflect and disperse the stress when the external force was loaded on the GFs. In this process, more stress would be consumed, which in turn led to an enhancement in compressive strength.

### 3.4 Ti-catalyzed graphitization mechanism

In this study, MD simulations were used to study the mechanism of Ti particle-catalyzed graphitization from the molecular level. ARMP and Ti were used as raw materials in the preparation of GFs. We chose a typical average molecule structure of the ARMP as a representative to simulate the Ti-catalyzed graphitization process.^17^ Due to the limitations of the current molecular simulation and the fact that no other substances were introduced in the heat treatment process of foaming process and carbonization. Therefore, the precursor heat treatment process was ignored and the ARMP molecules reacted directly with Ti at 2727 °C. In order to visualize and clearly understand the graphitization reaction catalyzed by Ti particles, 10 carbon atoms were selected for the study, and only the carbon ring formation and change processes were represented.

**Fig. 12:**
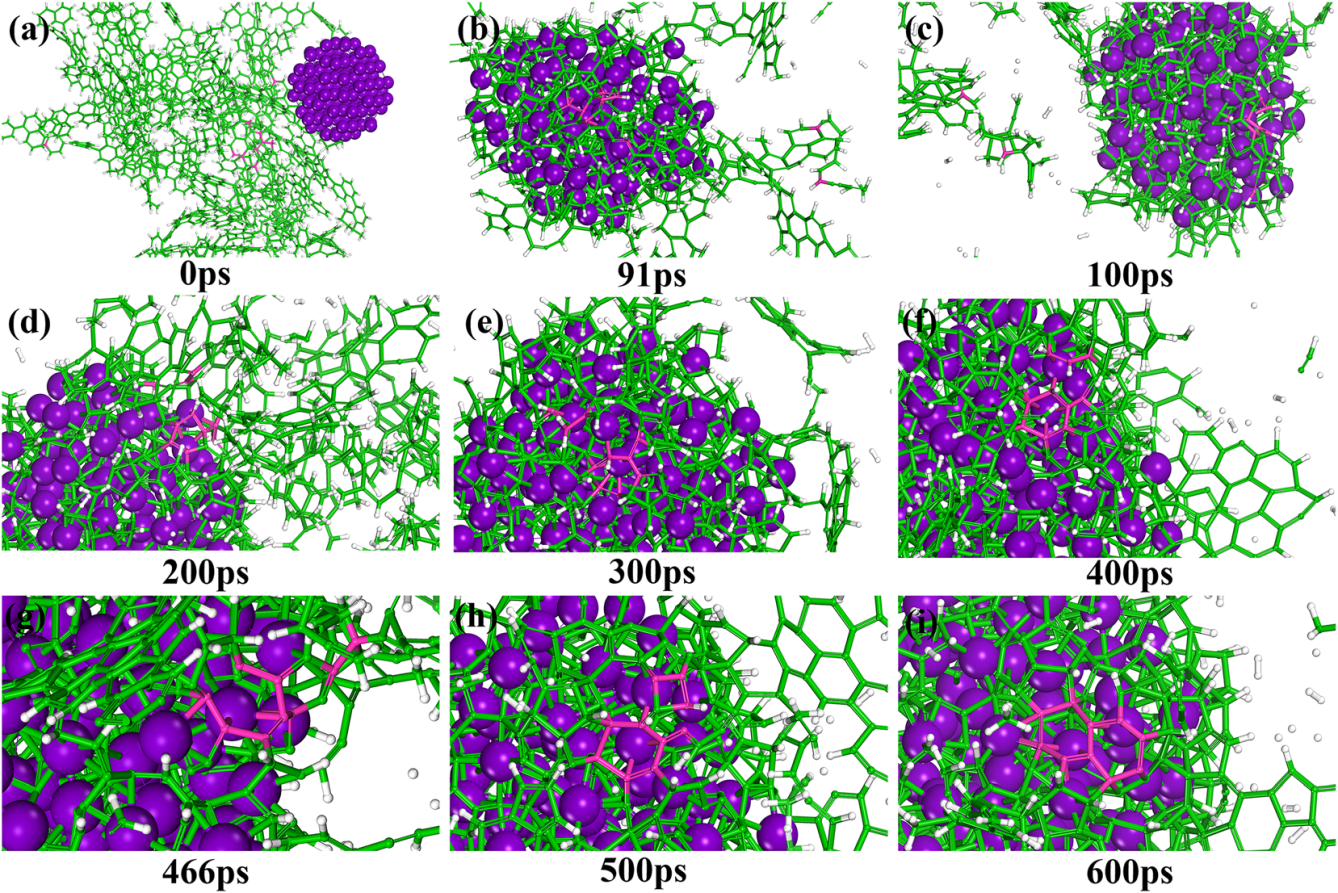
Snapshots showing the graphitization mechanisms of ARMP catalyzed by Ti particles (a–i). C atoms are shown in green (the selected C atoms are shown in pink), Ti in purple, and H in white.

**Fig. 13:**
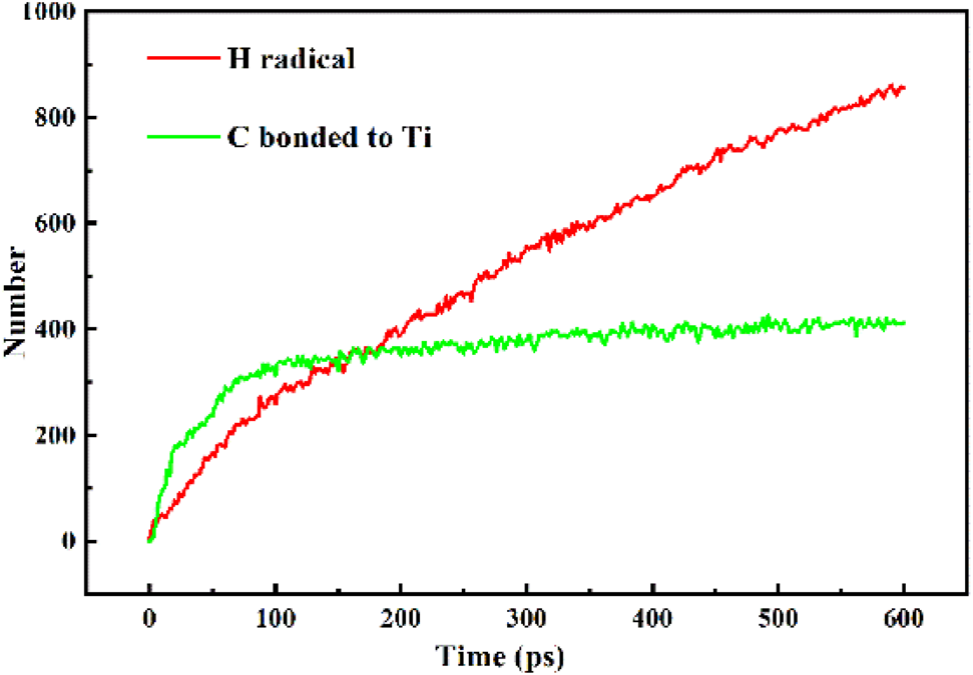
Evolution of H, and C bonded to Ti particle.

Fig. S5† displayed the process of Ti particles entering the molecular structure of the ARMP. As the reaction proceeded, ARMP molecules approached the Ti particle and gradually participated in the reaction with carbon. Firstly, the Ti atoms were observed to bond with the carbon atoms of the ARMP molecules, and the number of Ti–C bonds increased rapidly and stabilized, which also proved that the reaction produced a large amount of TiC (eqn (5)). In the practical process plenty of impurity atoms were removed during the carbonization process, and the graphitization process is also a further process of removing impurity atoms. During the simulation, a lot of H radicals were seen to be generated, which were constantly freed from the ARMP molecules, ensuring a more stable overall molecular structure during the graphitization process (Fig. 13). Subsequently, the distance between carbon atoms was gradually shortened due to the strong bonding between Ti and C in TiC, and as the graphitization process proceeded the previously generated Ti–C bonds were broken (Fig. 12g). Then, they decomposed to form Ti atoms and aromatic carbon atoms (eqn (6)). The TiC formation-decomposition process was repeated throughout the graphitization process maintaining the continuity of the catalytic process, and the number of Ti–C bonds tended to a stable value as displayed in Fig. 13. Between the newly decomposed carbon atoms formed C–C bonds combined with a new aromatic six-membered ring (Fig. 12h) (eqn (7)), and part of the carbon atoms of the six-membered ring remain bonded to Ti atoms. The remaining carbon atoms were assembled around the aromatic six-membered ring by the action of TiC to form two aromatic six-membered rings (Fig. 12i) (eqn (8)). Therefore, MD simulations confirmed that Ti contributed positively to the ARMP molecules with catalytic graphitization process, and revealed that its catalytic mechanism was consistent with the formation-decomposition mechanism of carbides.

5 Ti + C → TiC

6 TiC → Ti + C

7
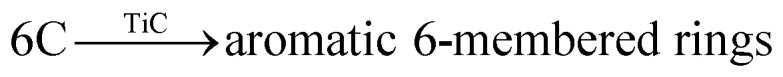
8
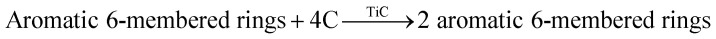


## 4. Conclusion

Light-weight GFs with high thermal conductivity and suitable compressive strength were prepared from an *in situ* Ti assisted catalytic graphitization approach by using ARMP as the precursor. The incorporation of Ti into the ARMP could significantly affect the microstructure and properties of GFs. The perfected structure of the GFs allows for easy and convenient thermal diffusivity, leading to improved thermal conductivity. GF prepared with the 11 wt% Ti addition has the highest thermal conductivity (60.8 W m^−1^ K^−1^) and moderate bulk density (0.36 g cm^−3^). *In situ* crystallization of TiC particles could partake loading, and disperse the stress, so they possessed better compressive strength. We have made exciting progress in the mechanism of catalytic graphitization of Ti-catalyzed ARMP by the ReaxFF method at the molecular level. The simulation demonstrated that the mechanism of Ti-catalyzed graphitization is inevitably related to the formation and breaking of Ti–C bonds. The formation of aromatic 6-membered rings was promoted by Ti particles. Ti-catalyzed ARMP graphitization resulted from the formation-decomposition mechanism of carbides.

## Conflicts of interest

There are no conflicts to declare.

## Supplementary Material

RA-013-D2RA06164C-s001

## References

[cit1] Liu H., Wu S., Tian N., Yan F., You C., Yang Y. (2020). J. Mater. Chem. A.

[cit2] Inagaki M., Qiu J., Guo Q. (2015). Carbon.

[cit3] Quintana J. M., Mower T. M. (2017). Mater. Des..

[cit4] Chai Y., Yang X. H., Zhao M., Chen Z. Y., Meng X. Z., Jin L. W., Zhang Q. L., Hu W. J. (2017). J. Heat Transfer.

[cit5] Kim J.-H., Jeong E., Lee Y.-S. (2015). J. Ind. Eng. Chem..

[cit6] Duan S., Wu X., Zeng K., Tao T., Huang Z., Fang M., Liu Y., Min X. (2020). Carbon.

[cit7] Li W., Feng L., Shi X., Wang Y. (2021). Adv. Eng. Mater..

[cit8] Tang R., Xu P., Dong J., Gui H., Zhang T., Ding Y., Murugadoss V., Naik N., Pan D., Huang M., Guo Z. (2022). Carbon.

[cit9] Huang Q., Xu Y., Guo Y., Zhang L., Hu Y., Qian J., Huang S. (2022). Carbon.

[cit10] Wang X., Dong A., Hu Y., Qian J., Huang S. (2020). Chem. Commun..

[cit11] Chen C., Kennel E. B., Stiller A. H., Stansberry P. G., Zondlo J. W. (2006). Carbon.

[cit12] Liu H., Li T., Huang T., Zhao X. (2015). J. Mater. Sci..

[cit13] Yadav A., Kumar R., Bhatia G., Verma G. L. (2011). Carbon.

[cit14] Focke W. W., Badenhorst H., Ramjee S., Kruger H. J., Van Schalkwyk R., Rand B. (2014). Carbon.

[cit15] Li W., Zhang H., Xia L. (2015). J. Porous Mater..

[cit16] Mochida I., Shimizu K., Korai Y., Otsuka H., Sakai Y., Fujiyama S. (1990). Carbon.

[cit17] Klett J., Hardy R., Romine E., Walls C., Burchell T. (2000). Carbon.

[cit18] Thambiliyagodage C. J., Ulrich S., Araujo P. T., Bakker M. G. (2018). Carbon.

[cit19] Aboul-Enein A. A., Awadallah A. E. (2018). Chem. Eng. J..

[cit20] Lin Q., Feng Z., Liu Z., Guo Q., Hu Z., He L., Ye H. (2015). Carbon.

[cit21] Liu Y., Liu Q., Gu J., Kang D., Zhou F., Zhang W., Wu Y., Zhang D. (2013). Carbon.

[cit22] Rastegar H., Bavand-vandchali M., Nemati A., Golestani-Fard F. (2018). Phys. E.

[cit23] Liu T., Liu E., Ding R., Luo Z., Hu T., Li Z. (2015). Electrochim. Acta.

[cit24] Nettelroth D., Schwarz H.-C., Burblies N., Guschanski N., Behrens P. (2016). Phys. Status Solidi A.

[cit25] Fu H., Zhao P., Xu S., Cheng G., Li Z., Li Y., Li K., Ma S. (2019). Chem. Eng. J..

[cit26] Mochida I., Ohtsubo R., Takeshita K., Marsh H. (1980). Carbon.

[cit27] Baraniecki C., Pinchbeck P. H., Pickering F. B. (1969). Carbon.

[cit28] Qiu H., Song Y., Liu L., Zhai G., Shi J. (2003). Carbon.

[cit29] Marsh H., Warburton A. P. (1976). Carbon.

[cit30] Murty H. N., Biederman D. L., Heintz E. A. (1973). Carbon.

[cit31] Rodriguez-Manzo J. A., Pham-Huu C., Banhart F. (2011). ACS Nano.

[cit32] Xiao S.-Y., Liu H.-B., Chen Y.-F., Hu C.-C. (2010). J. Chin. Ceram. Soc..

[cit33] Ōya A., Marsh H. (1982). J. Mater. Sci..

[cit34] Centeno A., Blanco C., Santamaría R., Granda M., Menéndez R. (2012). Carbon.

[cit35] Zhang G., Guo Q., Liu Z., Yao L., Liu L., Li J., Chen J. (2002). J. Nucl. Mater..

[cit36] Tuckerman M. E., Martyna G. J. (1999). J. Phys. Chem. B.

[cit37] Tang L., Mao Q., You Z., Yao Z., Zhu X., Zhong Q., Xiao J. (2022). Carbon.

[cit38] Liu D., Kong Q.-Q., Jia H., Xie L.-J., Chen J., Tao Z., Wang Z., Jiang D., Chen C.-M. (2021). Carbon.

[cit39] Feng W., Qin M., Lv P., Li J., Feng Y. (2014). Carbon.

[cit40] Sadezky A., Muckenhuber H., Grothe H., Niessner R., Pöschl U. (2005). Carbon.

[cit41] Du Y., Wang C. A., Che D., Mathews J. P. (2022). Fuel.

[cit42] Ewen J. P., Latorre C. A., Gattinoni C., Khajeh A., Moore J. D., Remias J. E., Martini A., Dini D. (2020). J. Phys. Chem. C.

[cit43] Nosé S. (1991). Prog. Theor. Phys. Suppl..

[cit44] Calvo M., García R., Arenillas A., Suárez I., Moinelo S. R. (2005). Fuel.

[cit45] Chen K., Zhang H., Ibrahim U.-K., Xue W., Liu H., Guo A. (2019). Fuel.

[cit46] Andreoli S., Eser S. (2020). Carbon.

[cit47] Vujković M., Bajuk-Bogdanović D., Matović L., Stojmenović M., Mentus S. (2018). Carbon.

[cit48] Li S., Tian Y., Zhong Y., Yan X., Song Y., Guo Q., Shi J., Liu L. (2011). Carbon.

[cit49] Fanjul F. (2002). Fuel.

[cit50] Guan T., Zhang G., Zhao J., Wang J., Li K. (2019). Fuel.

[cit51] Zhang G., Guan T., Wu J., Wang J., Wang N., Li K. (2021). Fuel.

[cit52] Molina-Jordá J. M. (2018). Carbon.

[cit53] Klett J. W., McMillan A. D., Gallego N. C., Walls C. A. (2004). J. Mater. Sci..

[cit54] Gallego N. C., Klett J. W. (2003). Carbon.

[cit55] Wang D. W., Li F., Liu M., Lu G. Q., Cheng H. M. (2008). Angew. Chem., Int. Ed. Engl..

[cit56] Nan C. W., Clarke D. R. (1996). Acta Mater..

[cit57] Li W. Q., Zhang H. B., Xiong X., Xiao F. (2010). Mater. Sci. Eng., A.

[cit58] Li W. Q., Zhang H. B., Xiong X., Xiao F. (2011). Mater. Sci. Eng., A.

[cit59] Li W. Q., Zhang H. B., Xiong X., Xiao F. (2010). Mater. Sci. Eng., A.

[cit60] Li S., Song Y., Song Y., Shi J., Liu L., Wei X., Guo Q. (2007). Carbon.

[cit61] Zhang T., Yuan H., Yang S. (2020). Eng. Fract. Mech..

[cit62] Zhu J., Wang X., Guo L., Wang Y., Wang Y., Yu M., Lau K.-t. (2007). Carbon.

