# Supplementary material for: In situ Ti assisted graphitization approach for the preparation of graphite foam with light weight and high thermal conductivity
Source: RSC Adv. 2023 Feb 20;13(9):6075–86. doi: 10.1039/d2ra06164c (PMC9939979; doi:10.1039/d2ra06164c)
Supplement: RA-013-D2RA06164C-s001 [file RA-013-D2RA06164C-s001.pdf]

Electronic Supplementary Material (ESI) for RSC Advances.
This journal is © The Royal Society of Chemistry 2023

Supporting information

In-situ Ti assisted graphitization approach for the preparation of graphite foam with lightweight and high thermal conductivity

Xing Guo[a,b], Yaxiong Liu[a,b], Xiaodong Tian[a], Zechao Tao[a,b,c], Xi Yan[a,b]*, Zhanjun Liu[a,b,c]*

[a] CAS Key Laboratory of Carbon Materials, Institute of Coal Chemistry, Chinese Academy of Sciences, Taiyuan, 030001, China

[b] Center of Materials Science and Optoelectronics Engineering, University of Chinese Academy of Sciences, Beijing 100049, China

[c] Dalian National Laboratory for Clean Energy, Dalian 116023, China

*Corresponding authors

E-mail addresses: yanxi@sxicc.ac.cn (Xi Yan)

(Zhanjun Liu)

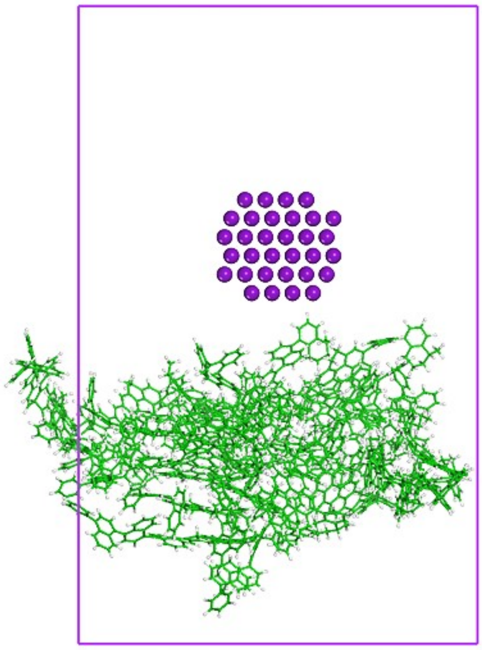

Fig.S1. The initial simulation cell of 20 ARMP molecules and 120 Ti atoms. C atoms shown in green, Ti in purple, and H in white.

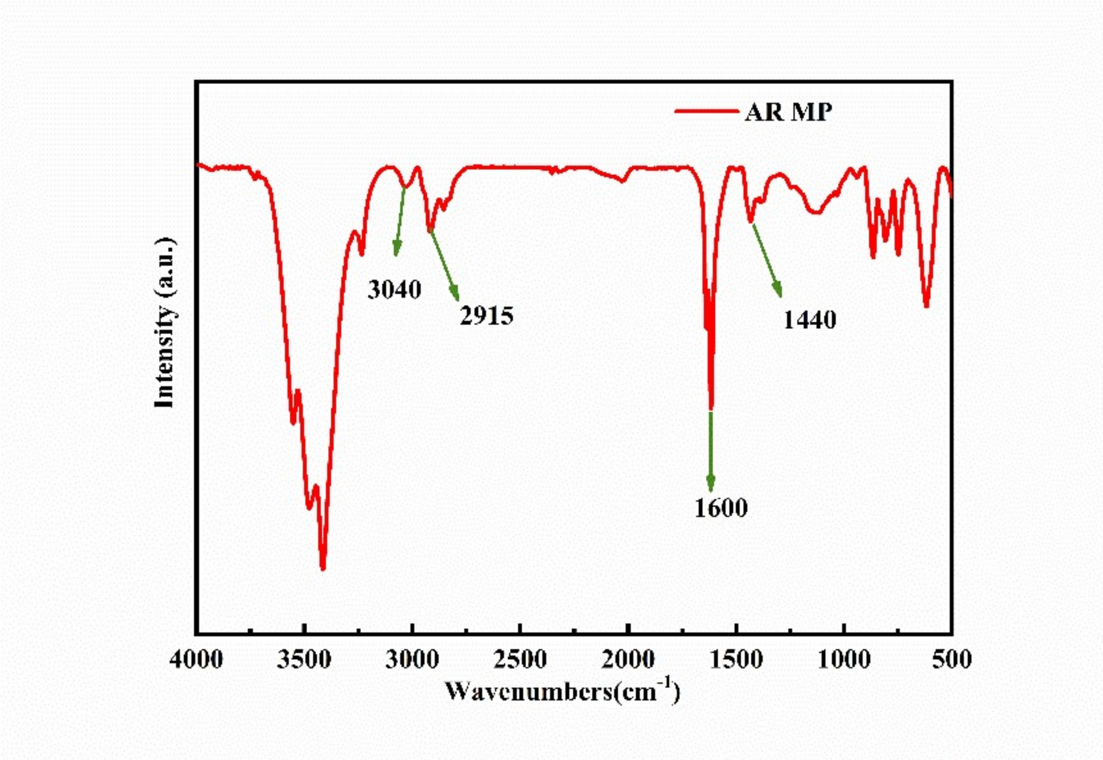

Fig.S2. The FTIR spectra of the ARMP.

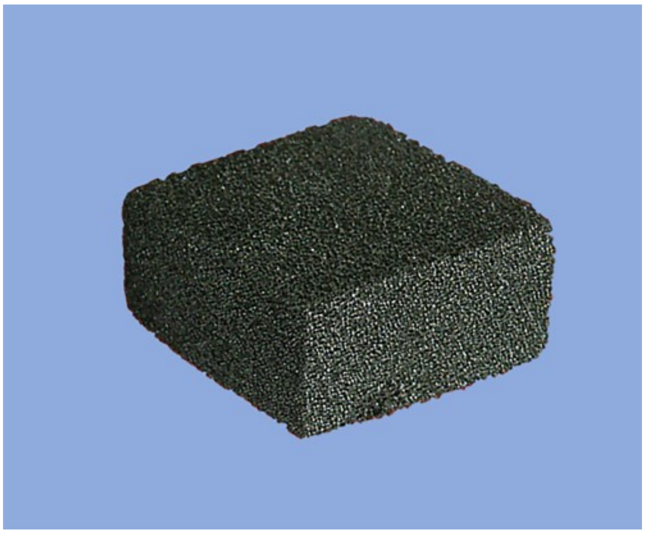

Fig.S3. The images of GF-11 taken from a camera

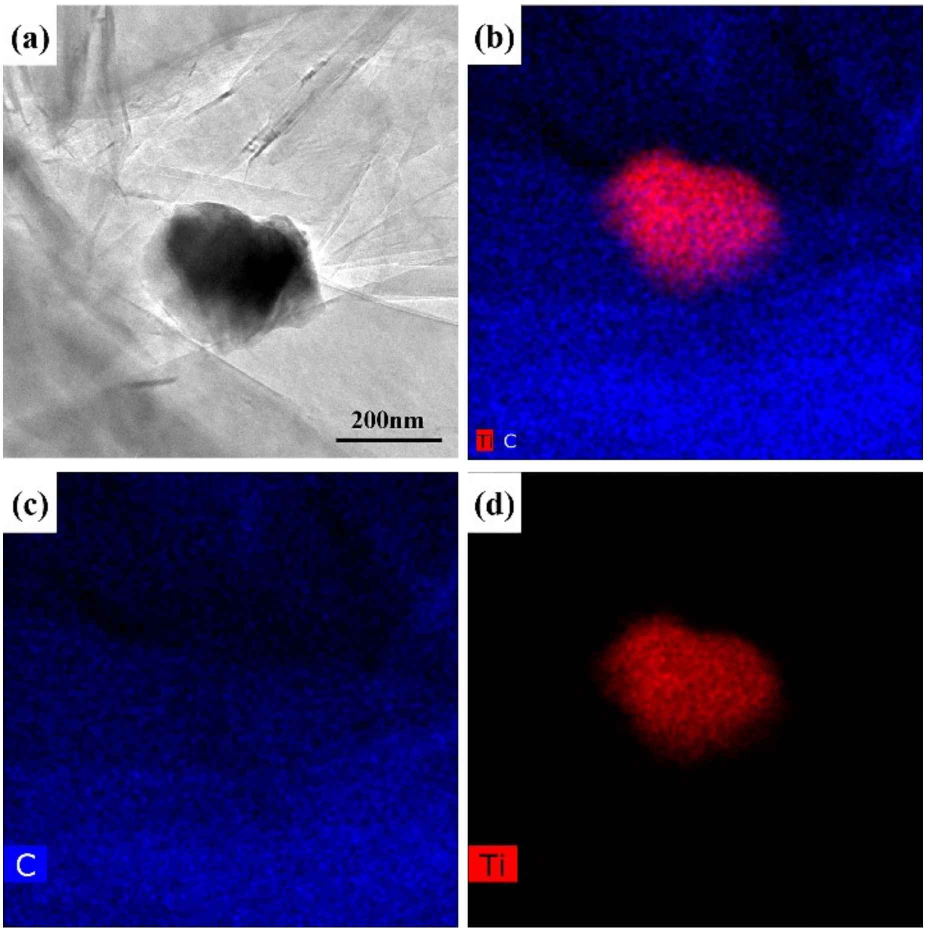

Fig.S4. EDS mapping analysis (a)-(d) of GF-11 from TEM.

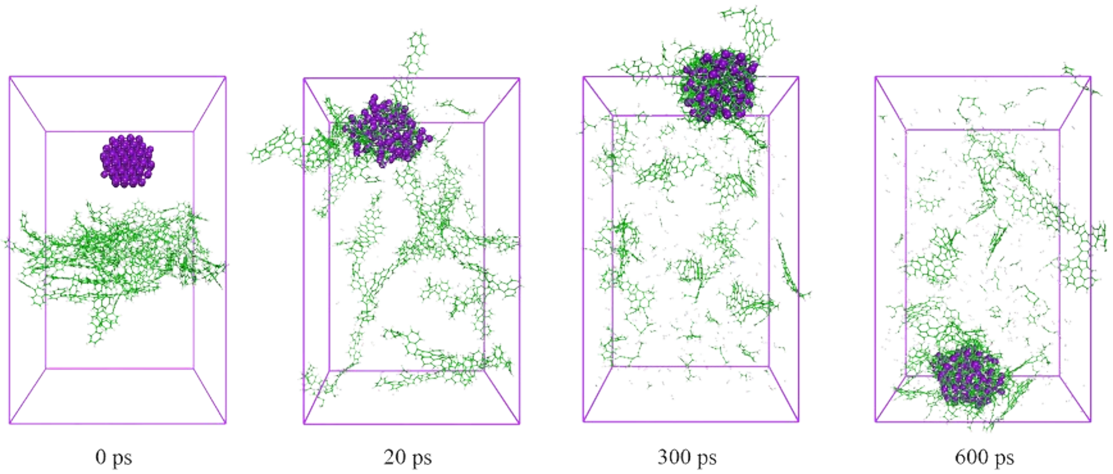

Fig.S5. Configurations of the model system in 600 ps ReaxFF molecular dynamics simulation.

Table S1. Specific heat (C_p) of GFs.

**Samples**	**C_p (J g^{-1} K^{-1})**
GF-0	0.713
GF-3	0.704
GF-5	0.698
GF-7	0.685
GF-11	0.617
GF-15	0.582

Table S2. Properties of mesophase pitch.

**Properties**	**Units**	**AR MP**
Softening point	℃	282
Toluene insoluble content	wt.%	55.0
Pyridine insoluble content	wt.%	51.5
Ash content	wt.%	0.03
Volatile content	wt.%	28.4
Mesophase content	wt.%	100

```
Reactive MD-force field: Ti/O/H
39       ! Number of general parameters
   50.0000 !Overcoordination parameter
    9.5469 !Overcoordination parameter
    1.6725 !Valency angle conjugation parameter
    1.7224 !Triple bond stabilisation parameter
    6.8702 !Triple bond stabilisation parameter
   60.4850 !C2-correction
    1.0588 !Undercoordination parameter
    4.6000 !Triple bond stabilisation parameter
   12.1176 !Undercoordination parameter
   13.3056 !Undercoordination parameter
  -63.5000 !Triple bond stabilization energy
    0.0000 !Lower Taper-radius
   10.0000 !Upper Taper-radius
    2.8793 !Not used
   33.8667 !Valency undercoordination
    6.0891 !Valency angle/lone pair parameter
    1.0563 !Valency angle
    2.0384 !Valency angle parameter
    6.1431 !Not used
    6.9290 !Double bond/angle parameter
    0.3989 !Double bond/angle parameter: overcoord
    3.9954 !Double bond/angle parameter: overcoord
   -2.4837 !Not used
    5.7796 !Torsion/BO parameter
   10.0000 !Torsion overcoordination
    1.9487 !Torsion overcoordination
   -1.2327 !Conjugation 0 (not used)
    2.1645 !Conjugation
    1.5591 !vdWaals shielding
    0.0100 !Cutoff for bond order (*100)
    1.7602 !Valency angle conjugation parameter
    0.6991 !Overcoordination parameter
   50.0000 !Overcoordination parameter
    1.8512 !Valency/lone pair parameter
    0.5000 !Not used
   20.0000 !Not used
    5.0000 !Molecular energy (not used)
    0.0000 !Molecular energy (not used)
    0.7903 !Valency angle conjugation parameter
 14    ! Nr of atoms; cov.r; valency;a.m;Rvdw;Evdw;gammaEEM;cov.r2;#
            alfa;gammavdW;valency;Eunder;Eover;chiEEM;etaEEM;n.u.
            cov r3;Elp;Heat inc.;n.u.;n.u.;n.u.;n.u.
```

ov/un;val1;n.u.;val3,vval4

C	1.3674	4.0000	12.0000	2.0453	0.1444	0.8485	1.1706	4.0000
	9.0000	1.5000	4.0000	30.0000	79.5548	4.8446	7.0000	0.0000
	1.1168	0.0000	181.0000	14.2732	24.4406	6.7313	0.8563	0.0000
	-4.1021	5.0000	1.0564	4.0000	2.9663	0.0000	0.0000	0.0000
H	0.8930	1.0000	1.0080	1.3550	0.0930	0.8203	-0.1000	1.0000
	8.2230	33.2894	1.0000	0.0000	121.1250	3.7248	9.6093	1.0000
	-0.1000	0.0000	55.1878	3.0408	2.4197	0.0003	1.0698	0.0000
	-19.4571	4.2733	1.0338	1.0000	2.8793	0.0000	0.0000	0.0000
O	1.2450	2.0000	15.9990	2.3890	0.1000	1.0898	1.0548	6.0000
	9.7300	13.8449	4.0000	37.5000	116.0768	8.5000	8.3122	2.0000
	0.9049	0.4056	68.0152	3.5027	0.7640	0.0021	0.9745	0.0000
	-3.5500	2.9000	1.0493	4.0000	2.9225	0.0000	0.0000	0.0000
N	1.2333	3.0000	14.0000	1.9324	0.1376	0.7921	1.1748	5.0000
	10.0667	7.8431	4.0000	32.2482	100.0000	7.5795	6.3952	2.0000
	1.0433	27.4290	119.9837	1.9457	4.2874	3.4869	0.9745	0.0000
	-4.3875	2.6192	1.0183	4.0000	2.8793	0.0000	0.0000	0.0000
S	1.9405	2.0000	32.0600	2.0677	0.2099	1.0336	1.5479	6.0000
	9.9575	4.9055	4.0000	52.9998	112.1416	5.7824	8.2545	2.0000
	1.4601	9.7177	71.1843	5.7487	23.2859	12.7147	0.9745	0.0000
	-11.0000	2.7466	1.0338	6.2998	2.8793	0.0000	0.0000	0.0000
Mg	1.8315	2.0000	24.3050	2.2464	0.1806	0.5020	1.0000	2.0000
	10.9186	27.1205	3.0000	38.0000	0.0000	0.9499	5.6130	0.0000
	-1.3000	0.0000	127.9160	49.9248	0.3370	0.0000	0.0000	0.0000
	-1.0823	2.3663	1.0564	6.0000	2.9663	0.0000	0.0000	0.0000
P	1.5994	3.0000	30.9738	1.7000	0.1743	1.0000	1.3000	5.0000
	9.1909	14.9482	5.0000	0.0000	0.0000	1.6676	7.0946	0.0000
	-1.0000	25.0000	125.6300	0.2187	21.4305	15.1425	0.0000	0.0000
	-3.9294	3.4831	1.0338	5.0000	2.8793	0.0000	0.0000	0.0000
Na	0.0001	1.0000	22.9898	2.6441	0.2588	0.8011	-1.0000	1.0000
	9.0003	2.5000	1.0000	0.0000	0.0000	-3.4731	8.6438	0.0000
	-1.0000	0.0000	23.0445	100.0000	1.0000	0.0000	0.8563	0.0000
	-4.1479	3.9900	1.0338	8.0000	2.5791	0.0000	0.0000	0.0000
Ti	2.0254	4.0000	47.8800	2.2105	0.1574	0.6311	0.1000	4.0000
	12.7041	16.6482	4.0000	0.1000	0.0000	-1.3647	6.8406	0.0000
	-1.0000	0.0000	143.1770	27.6505	-0.0753	0.0064	0.8563	0.0000
	-15.0000	3.8359	1.0338	12.0000	2.2632	0.0000	0.0000	0.0000
Cl	0.0014	1.0000	35.4500	2.0560	0.4000	0.3640	-1.0000	7.0000
	9.8895	10.1330	1.0000	0.0000	0.0000	7.8039	9.5196	2.0000
	-1.0000	0.0100	35.1770	6.2293	5.2294	0.1542	0.8563	0.0000
	-10.2080	2.9867	1.0338	6.2998	2.5791	0.0000	0.0000	0.0000
F	1.1846	1.0000	18.9984	1.7922	0.1267	0.4038	-0.1000	7.0000
	10.3184	7.5000	1.0000	9.2533	0.2000	9.3891	6.5612	2.0000
	-1.0000	3.5571	18.0000	6.9821	4.1799	1.0561	0.0000	0.0000

```
   -7.3000   2.6656   1.0493   4.0000   2.9225   0.0000   0.0000   0.0000
K   0.0001   1.0000  39.0983   2.6480   0.1676   0.3343  -1.0000   1.0000
    9.0047   2.5000   1.0000   0.0000   0.0000  -5.0000  10.4546   0.0000
   -1.0000   0.0000  23.0445 100.0000   1.0000   0.0000   0.8563   0.0000
   -2.5000   3.9900   1.0338   8.0000   2.5791   0.0000   0.0000   0.0000
Li  0.0001   1.0000   6.9410   2.6000   0.0865   0.8380  -0.1000   1.0000
    9.6984   1.4649   1.0000   0.0000   0.0000  -4.0561   9.7698   0.0000
   -1.0000   0.0000  37.5000   5.4409   6.9107   0.1973   0.8563   0.0000
  -24.7916   2.2989   1.0338   1.0000   2.8103   1.3000   0.2000  13.0000
X  -0.1000   2.0000   1.0080   2.0000   0.0000   0.0100  -0.1000   6.0000
   10.0000   2.5000   4.0000   0.0000   0.0000   5.0000 9999.999   0.0000
   -0.1000   0.0000  -2.3700   8.7410  13.3640   0.6690   0.9745   0.0000
  -11.0000   2.7466   1.0338   2.0000   2.8793   0.0000   0.0000   0.0000
71      ! Nr of bonds; Edis1;LPpen;n.u.;pbe1;pbo5;13corr;pbo6
                          pbe2;pbo3;pbo4;Etrip;pbo1;pbo2;ovcorr
  1  1  80.8865 107.9944  52.0636   0.5218  -0.3636   1.0000  34.9876   0.7769
          6.1244  -0.1693   8.0804   1.0000  -0.0586   8.1850   1.0000   0.0000
  1  2 180.6309   0.0000   0.0000  -0.4794   0.0000   1.0000   6.0000   0.6281
         12.2202   1.0000   0.0000   1.0000  -0.0670   6.8158   0.0000   0.0000
  2  2 153.3934   0.0000   0.0000  -0.4600   0.0000   1.0000   6.0000   0.7300
          6.2500   1.0000   0.0000   1.0000  -0.0790   6.0552   0.0000   0.0000
  1  3 163.3110  83.9973  54.4316  -0.5220  -0.3123   1.0000  10.2503   1.0000
          0.3553  -0.3757   7.0000   1.0000  -0.1331   4.6021   0.0000   0.0000
  3  3 142.2858 145.0000  50.8293   0.2506  -0.1000   1.0000  29.7503   0.6051
          0.3451  -0.1055   9.0000   1.0000  -0.1225   5.5000   1.0000   0.0000
  1  4 134.1215 140.2179  79.9745   0.0163  -0.1428   1.0000  27.0617   0.2000
          0.1387  -0.3681   7.1611   1.0000  -0.1000   5.0825   1.0000   0.0000
  3  4 130.8596 169.4551  40.0000   0.3837  -0.1639   1.0000  35.0000   0.2000
          1.0000  -0.3579   7.0004   1.0000  -0.1193   6.8773   1.0000   0.0000
  4  4 157.9384  82.5526 152.5336   0.4010  -0.1034   1.0000  12.4261   0.5828
          0.1578  -0.1509  11.9186   1.0000  -0.0861   5.4271   1.0000   0.0000
  2  3 160.0000   0.0000   0.0000  -0.5725   0.0000   1.0000   6.0000   0.5626
          1.1150   1.0000   0.0000   0.0000  -0.0920   4.2790   0.0000   0.0000
  2  4 185.3171   0.0000   0.0000  -0.3689   0.0000   1.0000   6.0000   0.2854
          7.6517   1.0000   0.0000   1.0000  -0.0408   6.0255   0.0000   0.0000
  1  5 128.7959  56.4134  39.0716   0.0688  -0.4463   1.0000  31.1766   0.4530
          0.1955  -0.3587   6.2148   1.0000  -0.0770   6.6386   1.0000   0.0000
  2  5 136.1049   0.0000   0.0000  -0.4669   0.0000   1.0000   6.0000   0.3803
         10.5730   1.0000   0.0000   1.0000  -0.1000   7.0000   1.0000   0.0000
  3  5 135.6998 220.0000  40.0000   0.5848  -0.2406   1.0000  22.1005   0.2335
          0.7069  -0.2681   8.3465   1.0000  -0.0922   5.4651   1.0000   0.0000
  4  5   0.0000   0.0000   0.0000   0.5000  -0.2000   1.0000  40.0000   0.3000
          0.4000  -0.2500   9.0000   1.0000  -0.1000   6.0000   1.0000   0.0000
  5  5   0.0000   0.0000   0.0000   0.2500  -0.5000   1.0000  20.0000   1.0000
```

		0.2500	-0.2500	10.0000	1.0000	-0.1000	8.0000	1.0000	0.0000
2	6	58.6896	0.0000	0.0000	-0.0203	-0.1418	1.0000	13.1260	0.0230
		8.2136	-0.1310	0.0000	1.0000	-0.2692	6.4254	0.0000	24.4461
3	6	87.0227	0.0000	43.3991	0.0030	-0.3000	1.0000	36.0000	0.0250
		0.0087	-0.2500	12.0000	1.0000	-0.0439	6.6073	1.0000	24.4461
6	6	32.3808	0.0000	0.0000	-0.0076	-0.2000	0.0000	16.0000	0.2641
		4.8726	-0.2000	10.0000	1.0000	-0.0729	4.6319	0.0000	0.0000
1	7	110.0000	92.0000	0.0000	0.2171	-0.1418	1.0000	13.1260	0.6000
		0.3601	-0.1310	10.7257	1.0000	-0.0869	5.3302	1.0000	0.0000
2	7	0.1466	0.0000	0.0000	0.2250	-0.1418	1.0000	13.1260	0.6000
		0.3912	-0.1310	0.0000	1.0000	-0.1029	9.3302	0.0000	0.0000
3	7	201.0058	194.1410	0.0000	1.0000	-0.5000	1.0000	25.0000	0.4873
		0.4358	-0.1571	15.8745	1.0000	-0.2431	6.3823	1.0000	0.0000
4	7	130.0000	0.0000	0.0000	0.2171	-0.1418	1.0000	13.1260	0.6000
		0.3601	-0.1310	10.7257	1.0000	-0.0869	5.3302	1.0000	0.0000
6	7	0.1000	0.0000	0.0000	0.2500	-0.5000	1.0000	35.0000	0.6000
		0.5000	-0.5000	20.0000	1.0000	-0.2000	10.0000	1.0000	0.0000
7	7	0.0000	0.0000	0.0000	0.2171	-0.5000	1.0000	35.0000	0.6000
		0.5000	-0.5000	20.0000	1.0000	-0.2000	10.0000	1.0000	0.0000
1	8	0.0000	0.0000	0.0000	0.5000	-0.3000	1.0000	16.0000	0.5000
		0.5000	-0.2500	15.0000	1.0000	-0.1000	9.0000	0.0000	0.0000
2	8	26.7569	0.0000	0.0000	1.0000	-0.3000	1.0000	36.0000	0.0100
		0.5785	-0.3500	25.0000	1.0000	-0.2601	6.6137	1.0000	0.0000
3	8	27.9718	0.0000	0.0000	0.0437	-0.3000	1.0000	36.0000	0.0100
		19.6220	-0.3500	25.0000	1.0000	-0.1279	7.3318	1.0000	0.0000
4	8	0.0000	0.0000	0.0000	-1.0000	-0.3000	1.0000	36.0000	0.7000
		10.1151	-0.3500	25.0000	1.0000	-0.1053	8.2003	1.0000	0.0000
5	8	0.0000	0.0000	0.0000	-1.0000	-0.3000	1.0000	36.0000	0.7000
		10.1151	-0.3500	25.0000	1.0000	-0.1053	8.2003	1.0000	0.0000
6	8	0.0000	0.0000	0.0000	0.2500	-0.5000	1.0000	35.0000	0.6000
		0.5000	-0.5000	20.0000	1.0000	-0.2000	10.0000	1.0000	0.0000
7	8	0.0000	0.0000	0.0000	0.2500	-0.5000	1.0000	35.0000	0.6000
		0.5000	-0.5000	20.0000	1.0000	-0.2000	10.0000	1.0000	0.0000
8	8	0.0000	0.0000	0.0000	-0.7273	0.3000	0.0000	25.0000	0.1919
		6.6441	-0.4000	12.0000	1.0000	-0.0345	5.0063	0.0000	0.0000
4	6	50.0000	10.0901	0.0000	-1.0000	-0.3000	1.0000	36.0000	0.7058
		0.8567	-0.3487	17.4990	1.0000	-0.0794	8.2232	1.0000	0.0000
1	9	135.3583	0.0000	0.0000	1.0000	-0.3000	0.0000	36.0000	0.0174
		0.0550	-0.2818	16.1571	1.0000	-0.1093	6.6103	0.0000	0.0000
2	9	0.0000	0.0000	0.0000	-0.2872	-0.3000	1.0000	36.0000	0.0082
		1.7973	-0.2500	20.0000	1.0000	-0.2578	6.5219	1.0000	0.0000
3	9	130.5629	37.6984	0.0000	0.9228	-0.3000	0.0000	36.0000	0.0850
		0.1150	-0.2818	16.1571	1.0000	-0.1343	6.8264	0.0000	0.0000
4	9	0.0000	0.0000	0.0000	-0.2872	-0.3000	1.0000	36.0000	0.0082

		1.7973	-0.2500	20.0000	1.0000	-0.2578	6.5219	1.0000	0.0000
5	9	0.0000	0.0000	0.0000	-0.2872	-0.3000	1.0000	36.0000	0.0082
		1.7973	-0.2500	20.0000	1.0000	-0.2578	6.5219	1.0000	0.0000
6	9	0.0000	0.0000	0.0000	-0.2872	-0.3000	1.0000	36.0000	0.0082
		1.7973	-0.2500	20.0000	1.0000	-0.2578	6.5219	1.0000	0.0000
7	9	0.0000	0.0000	0.0000	-0.2872	-0.3000	1.0000	36.0000	0.0082
		1.7973	-0.2500	20.0000	1.0000	-0.2578	6.5219	1.0000	0.0000
8	9	0.1000	0.0000	0.0000	0.2500	-0.5000	1.0000	35.0000	0.6000
		0.5000	-0.5000	20.0000	1.0000	-0.2000	10.0000	1.0000	0.0000
9	9	80.1930	0.0000	0.0000	-0.8469	-0.2000	0.0000	16.0000	0.2022
		0.7528	-0.1924	14.9725	1.0000	-0.0885	5.0000	0.0000	0.0000
1	10	213.3489	0.0000	0.0000	-0.5450	-0.5000	1.0000	35.0000	0.7833
		5.4257	-0.2500	15.0000	1.0000	-0.0827	5.9023	1.0000	0.0000
2	10	150.6697	0.0000	0.0000	-0.6499	-0.2000	0.0000	16.0000	0.8645
		3.8414	-0.2000	15.0000	1.0000	-0.2000	6.8063	0.0000	0.0000
3	10	0.0000	0.0000	0.0000	0.5000	-0.2000	0.0000	16.0000	0.5000
		1.0001	-0.2000	15.0000	1.0000	-0.1000	15.0000	0.0000	0.0000
10	10	148.6765	0.0000	0.0000	0.7040	-0.3500	0.0000	25.0000	0.9428
		-0.3546	-0.2500	15.0000	1.0000	-0.1329	7.0417	0.0000	0.0000
4	10	0.0000	0.0000	0.0000	0.5000	-0.2000	0.0000	16.0000	0.5000
		1.0001	-0.2000	15.0000	1.0000	-0.1000	10.0000	0.0000	0.0000
5	10	0.0000	0.0000	0.0000	0.5000	-0.2000	0.0000	16.0000	0.5000
		1.0001	-0.2000	15.0000	1.0000	-0.1000	10.0000	0.0000	0.0000
6	10	0.0000	0.0000	0.0000	0.5000	-0.2000	0.0000	16.0000	0.5000
		1.0001	-0.2000	15.0000	1.0000	-0.1000	10.0000	0.0000	0.0000
7	10	0.0000	0.0000	0.0000	0.5000	-0.2000	0.0000	16.0000	0.5000
		1.0001	-0.2000	15.0000	1.0000	-0.1000	10.0000	0.0000	0.0000
8	10	0.0000	0.0000	0.0000	0.5000	-0.2000	0.0000	16.0000	0.5000
		1.0001	-0.2000	15.0000	1.0000	-0.1000	10.0000	0.0000	0.0000
9	10	0.0000	0.0000	0.0000	-0.0830	-0.2000	0.0000	16.0000	0.5483
		0.1065	-0.2000	15.0000	1.0000	-0.0553	8.5063	0.0000	0.0000
1	11	237.8781	0.0000	0.0000	-0.7438	-0.5000	1.0000	35.0000	1.0460
		3.6661	-0.2500	15.0000	1.0000	-0.0800	5.4719	1.0000	0.0000
2	11	154.6080	0.0000	0.0000	-0.1948	-0.2000	0.0000	16.0000	0.1676
		16.3699	-0.2000	15.0000	1.0000	-0.2265	7.1308	0.0000	0.0000
3	11	0.0000	0.0000	0.0000	-0.4643	0.0000	1.0000	6.0000	0.6151
		12.3710	1.0000	0.0000	1.0000	-0.1008	8.5980	0.0000	0.0000
4	11	0.0000	0.0000	0.0000	-0.4643	0.0000	1.0000	6.0000	0.6151
		12.3710	1.0000	0.0000	1.0000	-0.1008	8.5980	0.0000	0.0000
5	11	0.0000	0.0000	0.0000	-0.4643	0.0000	1.0000	6.0000	0.6151
		12.3710	1.0000	0.0000	1.0000	-0.1008	8.5980	0.0000	0.0000
11	11	109.0438	0.0000	0.0000	0.6382	-0.3500	1.0000	25.0000	1.1695
		0.1254	-0.2500	15.0000	1.0000	-0.1062	5.9666	1.0000	0.0000
9	11	194.0978	0.0000	0.0000	-0.9855	-0.2000	0.0000	16.0000	0.4118

```
         1.0000  -0.2000  15.0000   1.0000  -0.0715   6.0975   0.0000   0.0000
  1 12   0.0000   0.0000   0.0000   0.5000  -0.3000   1.0000  16.0000   0.5000
         0.5000  -0.2500  15.0000   1.0000  -0.1000   9.0000   0.0000   0.0000
  2 12   0.0000   0.0000   0.0000  -1.0000  -0.3000   1.0000  36.0000   0.7000
        10.1151  -0.3500  25.0000   1.0000  -0.1053   8.2003   1.0000   0.0000
  3 12  22.6146   0.0000  43.0000   0.6651  -0.3000   1.0000  36.0000   1.0000
         0.9166  -0.3500  25.0000   1.0000  -0.0583   7.3861   1.0000   0.0000
 10 12  18.4962   0.0000   0.0000  -0.3256  -0.2000   0.0000  16.0000   0.0900
         2.4852  -0.2000  15.0000   1.0000  -0.0723   6.3893   0.0000   0.0000
 12 12   0.0000   0.0000   0.0000   0.3272   0.3000   0.0000  25.0000   0.5944
         0.9915  -0.4000  12.0000   1.0000  -0.0517   4.5075   0.0000   0.0000
  9 12   0.0000   0.0000   0.0000   0.5000  -0.3000   0.0000  16.0000   0.5000
         0.5000  -0.2500  15.0000   1.0000  -0.1000   9.0000   0.0000   0.0000
  2 13   0.0000   0.0000   0.0000   1.0000  -0.3000   1.0000  36.0000   0.0100
         0.3415  -0.3500  25.0000   1.0000  -0.2770   6.4396   1.0000   0.0000
  3 13  71.3512  -0.0200   0.0000   0.6715   0.3000   0.0000   6.0000   0.1621
         0.1284  -0.2500  11.9965   1.0000  -0.1026   5.8179   0.0000   0.0000
 10 13  39.6304   0.0000   0.0000  -0.6878  -0.3000   1.0000  16.0000   0.4891
         1.8957  -0.2500  15.0000   1.0000  -0.0471   5.0000   0.0000   0.0000
 13 13   0.0000   0.0000   0.0000   0.3228   0.3000   0.0000  26.0000   0.6003
         1.7161   0.0000  12.0000   1.0000  -0.1015   4.0000   0.0000   0.0000
  1 13   0.0000   0.0000   0.0000   0.3228   0.3000   0.0000  26.0000   0.6003
         1.7161   0.0000  12.0000   1.0000  -0.1015   4.0000   0.0000   0.0000
  9 13   0.0000   0.0000   0.0000   0.3228   0.3000   0.0000  26.0000   0.6003
         1.7161   0.0000  12.0000   1.0000  -0.1015   4.0000   0.0000   0.0000
37     ! Nr of off-diagonal terms; Ediss;Ro;gamma;rsigma;rpi;rpi2
  1  2   0.1200   1.3861   9.8561   1.1254  -1.0000  -1.0000
  2  3   0.0283   1.2885  10.9190   0.9215  -1.0000  -1.0000
  2  4   0.0687   1.5130  10.0094   0.9412  -1.0000  -1.0000
  1  3   0.1347   1.8343   9.7934   1.3139   1.1498   1.1039
  1  4   0.1447   1.8766   9.7990   1.3436   1.1885   1.1363
  3  4   0.1048   2.0003  10.1220   1.3173   1.1096   1.0206
  1  5   0.1408   1.8161   9.9393   1.7986   1.3021   1.4031
  2  5   0.0895   1.6239  10.0104   1.4640  -1.0000  -1.0000
  3  5   0.1962   1.7872  10.2319   1.4622   1.4025  -1.0000
  4  5   0.1505   1.9000  10.5104   1.8000   1.4000  -1.0000
  2  6   0.0100   1.6000  13.2979   1.8670  -1.0000  -1.0000
  3  6   0.0809   1.7000  11.4606   1.5177  -1.0000  -1.0000
  3  7   0.0534   1.7520  10.4281   1.8000   1.4498  -1.0000
  6  7   0.1801   1.8566   9.8498   0.1000  -1.0000  -1.0000
  1  8   0.2000   1.8500  11.0000  -1.0000  -1.0000  -1.0000
  2  8   0.1100   1.8410   9.1430   1.7735  -1.0000  -1.0000
  3  8   0.1536   1.6000  12.9050   1.6436  -1.0000  -1.0000
  2  9   0.1750   1.7939  13.5000   1.1000  -1.0000  -1.0000
```

3	9	0.1200	1.8000	10.5000	1.6526	1.4718	-1.0000
10	9	0.1368	2.2000	12.5000	1.8549	1.4718	-1.0000
1	9	0.2497	1.9709	11.2424	1.6312	-1.0000	-1.0000
1	10	0.1388	1.7970	11.9405	1.5598	-1.0000	-1.0000
2	10	0.1695	1.6156	9.7834	1.4740	-1.0000	-1.0000
3	10	0.1491	2.3500	10.1159	-1.0000	-1.0000	-1.0000
1	11	0.1046	1.6238	11.0397	1.5000	-1.0000	-1.0000
2	11	0.0553	1.7443	9.0006	1.3514	-1.0000	-1.0000
9	11	0.1049	1.6192	12.4193	1.7484	1.4718	-1.0000
1	12	0.2000	1.8500	11.0000	-1.0000	-1.0000	-1.0000
2	12	0.3000	1.5647	13.3924	-1.0000	-1.0000	-1.0000
3	12	0.1832	1.7503	12.6152	1.6986	-1.0000	-1.0000
10	12	0.2428	2.0950	11.0672	2.0182	-1.0000	-1.0000
9	12	0.2000	1.8500	11.0000	-1.0000	-1.0000	-1.0000
2	13	0.5000	1.4607	13.0000	-1.0000	-1.0000	-1.0000
3	13	0.0696	1.8520	10.0874	1.5559	-1.0000	1.0000
10	13	0.2331	1.6173	12.1979	1.7675	-1.0000	-1.0000
9	13	0.0200	1.8000	9.0000	-1.0000	-1.0000	-1.0000
8	9	0.4404	1.8207	12.5000	0.0100	-1.0000	-1.0000

114 ! Nr of angles;at1;at2;at3;Thetao,o;ka;kb;pv1;pv2

1	1	1	74.9085	44.7514	0.9144	0.0000	0.0050	0.3556	2.5715
1	1	2	68.0294	13.4722	5.5819	0.0000	0.6849	0.0000	1.0031
2	1	2	68.4575	22.1235	1.2937	0.0000	3.0000	0.0000	1.5009
1	2	2	0.0000	0.0000	6.0000	0.0000	0.0000	0.0000	1.0400
1	2	1	0.0000	7.5000	5.0000	0.0000	0.0000	0.0000	1.0400
2	2	2	0.0000	27.9213	5.8635	0.0000	0.0000	0.0000	1.0400
1	1	3	15.7798	9.0805	4.0304	0.0000	1.8785	70.0000	1.1737
2	1	3	65.0000	13.4505	1.8249	0.0000	1.5646	0.0000	1.2173
3	1	3	74.7266	45.0000	1.8020	-16.7178	2.6091	0.1000	2.3556
1	1	4	78.5538	21.4381	7.4715	0.0000	1.1046	50.0000	1.5275
3	1	4	73.9544	12.4661	7.0000	0.0000	1.1046	0.0000	1.1880
4	1	4	89.3168	20.2660	7.5000	0.0000	1.1046	0.0000	1.5403
2	1	4	74.2929	31.0883	2.6184	0.0000	0.1000	0.0000	1.0500
1	2	4	0.0000	0.0019	6.3000	0.0000	0.0000	0.0000	1.0400
1	3	1	76.7840	44.2266	0.9343	0.0000	1.3483	0.0000	1.8301
1	3	3	63.9120	17.1680	0.8751	0.0000	0.0693	50.9415	3.0000
1	3	2	79.6413	28.6488	0.3789	0.0000	1.6776	0.0000	1.0010
2	3	2	85.8000	9.8453	2.2720	0.0000	2.8635	0.0000	1.5800
2	3	3	79.5453	45.0000	2.1630	0.0000	3.0000	0.0000	1.2391
3	3	3	80.7324	30.4554	0.9953	0.0000	1.6310	50.0000	1.0783
1	3	4	82.4890	31.4554	0.9953	0.0000	3.0000	0.0000	1.0783
3	3	4	84.3637	31.4554	0.9953	0.0000	3.0000	0.0000	1.0783
4	3	4	89.7071	31.4554	0.9953	0.0000	3.0000	0.0000	1.1519
2	3	4	75.6201	18.7919	0.9833	0.0000	0.1000	0.0000	1.0500

1	4	1	81.4699	7.2318	1.2608	0.0000	3.0000	0.0000	1.2127
1	4	3	103.3204	33.0381	0.5787	0.0000	3.0000	0.0000	1.2127
1	4	4	50.0000	25.0250	4.7651	0.0000	3.0000	0.0000	1.2028
3	4	3	74.1978	42.1786	1.7845	-18.0069	3.0000	0.0000	1.2127
3	4	4	74.8600	43.7354	1.1572	-0.9193	3.0000	0.0000	1.2127
4	4	4	75.0538	14.8267	5.2794	0.0000	3.0000	0.0000	1.2127
1	4	2	68.2294	29.6576	1.0533	0.0000	0.3481	0.0000	1.5443
2	4	3	81.3686	40.0712	2.2396	0.0000	0.3481	0.0000	1.5443
2	4	4	83.0104	43.4766	1.5328	0.0000	0.3481	0.0000	1.5443
2	4	2	79.6336	17.7917	3.7832	0.0000	0.0222	0.0000	2.0238
1	2	3	0.0000	45.0000	3.0000	0.0000	1.0000	0.0000	1.0400
1	2	4	0.0000	0.0019	6.0000	0.0000	0.0000	0.0000	1.0400
1	2	5	0.0000	0.0019	6.0000	0.0000	0.0000	0.0000	1.0400
3	2	3	0.0000	15.0000	2.8900	0.0000	0.0000	0.0000	2.8774
3	2	4	0.0000	0.0019	6.0000	0.0000	0.0000	0.0000	1.0400
4	2	4	0.0000	0.0019	6.0000	0.0000	0.0000	0.0000	1.0400
2	2	3	0.0000	8.5744	3.0000	0.0000	0.0000	0.0000	1.0421
2	2	4	0.0000	0.0019	6.0000	0.0000	0.0000	0.0000	1.0400
1	1	5	74.4180	33.4273	1.7018	0.1463	0.5000	0.0000	1.6178
1	5	1	79.7037	28.2036	1.7073	0.1463	0.5000	0.0000	1.6453
2	1	5	63.3289	29.4225	2.1326	0.0000	0.5000	0.0000	3.0000
1	5	2	85.9449	38.3109	1.2492	0.0000	0.5000	0.0000	1.1000
1	5	5	85.6645	40.0000	2.9274	0.1463	0.5000	0.0000	1.3830
2	5	2	83.8555	5.1317	0.4377	0.0000	0.5000	0.0000	3.0000
2	5	5	97.0064	32.1121	2.0242	0.0000	0.5000	0.0000	2.8568
3	5	3	81.0926	30.2268	6.4132	-5.4471	2.5968	0.0000	3.0000
1	5	3	70.0000	35.0000	3.4223	0.0000	1.3550	0.0000	1.2002
1	3	5	57.3353	41.0012	1.0609	0.0000	1.3000	0.0000	3.0000
3	3	5	83.9753	31.0715	3.5590	0.0000	0.8161	0.0000	1.1776
2	3	5	89.8843	17.5000	3.3660	0.0000	2.0000	0.0000	2.0734
2	6	2	0.0000	49.8261	0.2093	0.0000	2.0870	0.0000	2.2895
2	2	6	0.0000	40.0366	3.1505	0.0000	1.1296	0.0000	1.1110
6	2	6	0.0000	0.5047	0.8000	0.0000	0.8933	0.0000	4.6650
2	6	6	0.0000	8.7037	0.0827	0.0000	3.5597	0.0000	1.1198
3	6	3	0.0000	9.2317	0.1000	0.0000	1.0000	0.0000	1.0920
6	3	6	0.0008	25.0000	8.0000	0.0000	1.0000	0.0000	3.0000
2	3	6	66.0423	5.0000	1.0000	0.0000	1.0000	0.0000	1.2500
2	6	3	0.0000	0.5000	0.1000	0.0000	1.0000	0.0000	3.0000
3	3	6	70.0000	20.0000	1.0000	0.0000	1.0000	0.0000	1.2500
3	7	3	90.0000	18.4167	0.6799	-8.0000	0.1310	0.0000	2.2321
2	3	7	72.6004	9.6150	0.8905	0.0000	3.5473	0.0000	1.0400
3	3	7	60.0000	40.0000	4.0000	0.0000	1.0000	0.0000	1.0400
3	2	7	0.0000	10.0000	1.0000	0.0000	1.0000	0.0000	1.0400
6	3	7	41.0995	3.2207	7.3523	0.0000	0.1101	0.0000	1.0947

7	3	7	62.1312	7.5931	0.1000	0.0000	0.5154	0.0000	2.1744
1	3	7	74.1394	8.5687	1.7132	0.0000	-0.6553	0.0000	2.2323
2	7	3	75.0000	25.0000	2.0000	0.0000	1.0000	0.0000	1.2500
3	7	7	70.0000	25.0000	2.0000	0.0000	1.0000	0.0000	1.2500
3	9	3	90.0000	30.4624	2.1468	0.0000	0.0500	0.0000	1.9485
9	3	9	90.0000	5.7486	5.0000	0.0000	2.0000	0.0000	1.1000
10	9	10	79.8775	32.7635	1.6551	0.0000	1.0510	0.0000	1.6270
9	10	9	28.5238	40.0000	5.0000	0.0000	2.0000	0.0000	1.1000
3	3	9	62.9344	15.0215	4.3743	0.0000	0.6168	0.0000	1.1673
3	9	9	33.7127	8.0623	3.4580	0.0000	0.0500	0.0000	2.6065
2	3	9	90.0000	9.7766	8.0000	0.0000	0.0505	0.0000	1.7257
1	3	9	47.6602	40.0000	9.9975	0.0000	1.6770	0.0000	1.5620
3	2	10	0.0000	0.5102	0.0100	0.0000	0.0000	0.0000	1.3399
11	1	11	77.8443	49.0744	5.9913	0.0000	0.7835	0.0000	2.3020
1	11	1	0.0000	19.9962	3.2299	0.0000	2.1012	0.0000	1.1537
1	11	11	0.0000	25.0000	1.0000	0.0000	1.0000	0.0000	1.0400
11	1	2	69.6421	10.0000	2.0000	0.0000	1.0000	0.0000	1.0400
1	9	1	79.0416	34.7981	2.4601	0.0000	4.4940	0.0000	4.0000
9	1	9	92.0437	39.9848	3.5810	0.0000	1.0016	0.0000	2.6458
1	9	3	8.4565	0.8893	1.9621	0.0000	2.1384	0.0000	3.6547
1	1	9	95.0000	35.4300	7.4753	0.0000	0.7434	0.0000	2.9506
1	9	11	67.4762	38.2782	4.9408	0.0000	0.1500	0.0000	1.8376
11	9	11	86.3838	39.7254	1.7723	0.0000	0.0500	0.0000	4.0000
9	11	9	20.0000	40.0000	5.0000	0.0000	0.6953	0.0000	1.5743
1	11	9	20.7615	38.6996	2.8535	0.0000	1.5402	0.0000	1.3906
3	9	11	120.0000	40.0000	1.1631	0.0000	2.0000	0.0000	1.1000
2	3	12	100.0000	1.0007	9.7740	0.0000	1.4276	0.0000	1.0000
12	3	12	98.5744	2.1499	1.6268	0.0000	3.7347	0.0000	2.8271
3	13	3	1.0000	6.7901	5.5000	0.0000	0.2366	0.0000	1.5936
2	3	13	100.0000	4.9629	8.0000	0.0000	0.1000	0.0000	1.0000
13	3	13	100.0000	8.0985	3.1295	0.0000	2.0000	0.0000	1.1755
3	13	10	54.0553	22.2701	8.0000	0.0000	2.3740	0.0000	2.0553
2	10	13	92.8098	7.7675	0.2250	0.0000	0.5870	0.0000	3.0000
9	3	13	76.5056	10.3180	0.9990	0.0000	0.7995	0.0000	1.0000
3	8	3	100.0000	40.0000	7.8728	0.0000	3.2930	0.0000	2.6836
2	3	8	82.0800	5.5605	8.0000	0.0000	1.4276	0.0000	1.6766
8	3	8	81.0000	4.7500	0.9000	0.0000	1.0000	0.0000	2.0000
8	3	9	81.3907	15.0000	3.0000	0.0000	1.5000	0.0000	1.8332
3	1	9	39.4706	40.0000	9.9991	0.0000	5.0000	0.0000	1.6832
9	11	11	90.0000	30.0000	5.0000	0.0000	1.0000	0.0000	1.1000
10	2	10	0.0000	5.0000	1.0000	0.0000	1.0000	0.0000	1.2500
10	10	10	0.0000	25.0000	1.0000	0.0000	1.0000	0.0000	1.2500
10	1	10	72.0864	22.4151	6.6934	0.0000	0.7328	0.0000	1.1557
1	10	1	0.0000	40.0000	4.8556	0.0000	2.5647	0.0000	1.4228

```
 1 10 10   0.0000  25.0000   1.0000   0.0000   1.0000   0.0000   1.0400
 2  1 10  74.6486  33.8387   7.4888   0.0000   2.7096   0.0000   1.0000
60    ! Nr of torsions;at1;at2;at3;at4;;V1;V2;V3;V2(BO);vconj;n.u;n
 1  1  1  1   2.1207  26.8713   0.5160  -9.0000  -2.8394   0.0000   0.0000
 1  1  1  2   1.4658  44.1251   0.4411  -5.3120  -2.1894   0.0000   0.0000
 2  1  1  2   1.4787  40.5128   0.4396  -5.2756  -3.0000   0.0000   0.0000
 1  1  1  3   0.9963  17.2365   0.1491  -2.5000  -1.0000   0.0000   0.0000
 2  1  1  3   1.5159  28.6602   0.7169  -7.5489  -3.0000   0.0000   0.0000
 3  1  1  3  -0.2000  23.6540  -1.0000  -5.9155  -1.1552   0.0000   0.0000
 1  1  3  1   1.8231  46.5696  -1.0000  -3.0536  -3.0000   0.0000   0.0000
 1  1  3  2   1.4836  80.0000   0.0363  -4.7349  -1.0000   0.0000   0.0000
 2  1  3  1   0.5983  49.5033   0.7210  -3.4046  -1.6880   0.0000   0.0000
 2  1  3  2  -0.2000  76.3511   1.0000  -4.0709  -1.0000   0.0000   0.0000
 1  1  3  3  -0.2000   5.0000  -1.0000  -3.6523  -2.9000   0.0000   0.0000
 2  1  3  3   2.5000  80.0000   1.0000  -2.6071  -3.0000   0.0000   0.0000
 3  1  3  1  -0.2000  80.0000  -1.0000  -3.6863  -3.0000   0.0000   0.0000
 3  1  3  2   2.5000  38.8954  -0.8368  -4.6681  -2.9000   0.0000   0.0000
 3  1  3  3  -0.2000  78.1766   0.0250  -2.8895  -3.0000   0.0000   0.0000
 1  3  3  1   2.5000   0.1000   1.0000  -2.6905  -2.7573   0.0000   0.0000
 1  3  3  2   0.5241  69.2788  -1.0000  -4.4539  -2.8081   0.0000   0.0000
 2  3  3  2   2.5000   0.1000  -0.4869  -2.8372  -1.0000   0.0000   0.0000
 1  3  3  3   2.5000   0.1000   1.0000  -3.4298  -1.0000   0.0000   0.0000
 2  3  3  3  -0.2000   0.1000  -1.0000  -3.5698  -1.0000   0.0000   0.0000
 3  3  3  3  -0.2000   0.1000   1.0000  -3.8409  -1.0000   0.0000   0.0000
 0  1  2  0   0.0000   0.0000   0.0000   0.0000   0.0000   0.0000   0.0000
 0  2  2  0   0.0000   0.0000   0.0000   0.0000   0.0000   0.0000   0.0000
 0  2  3  0   0.0000   0.1000   0.0200  -2.5415   0.0000   0.0000   0.0000
 0  1  1  0   0.0000  50.0000   0.3000  -4.0000  -2.0000   0.0000   0.0000
 0  1  4  0   1.7932 141.5515   0.9686  -4.2368  -1.9727   0.0000   0.0000
 0  2  4  0  -1.5000   0.1032   0.0100  -5.0965   0.0000   0.0000   0.0000
 0  3  4  0   1.1397  61.3225   0.5139  -3.8507  -3.0000   0.0000   0.0000
 0  4  4  0   0.7265  44.3155   1.0000  -4.4046  -2.0000   0.0000   0.0000
 4  1  4  4  -0.0949   8.7582   0.3310  -7.9430  -2.0000   0.0000   0.0000
 0  1  5  0   3.3423  30.3435   0.0365  -2.7171   0.0000   0.0000   0.0000
 0  5  5  0  -0.0555  -5.0000   0.1515  -2.2056   0.0000   0.0000   0.0000
 0  2  5  0   0.0000   0.0000   0.0000   0.0000   0.0000   0.0000   0.0000
 2  3  5  3   2.5000   2.5000   0.2237 -10.0000  -1.0000   0.0000   0.0000
 0  3  5  0  -2.5000  50.0000  -0.5000 -10.0000  -1.0000   0.0000   0.0000
 0  6  6  0   0.0000   0.0000   0.1200  -2.4426   0.0000   0.0000   0.0000
 0  2  6  0   0.0000   0.0000   0.1200  -2.4847   0.0000   0.0000   0.0000
 0  3  6  0   0.0000   0.0000   0.1200  -2.4703   0.0000   0.0000   0.0000
 1  1  1  7  -0.3232  14.3871   0.1823  -9.8682  -1.7255   0.0000   0.0000
 7  1  1  7  -0.1452  50.0000  -0.1915  -8.0773  -1.7255   0.0000   0.0000
 0  1  7  0   4.0000  45.8264   0.9000  -4.0000   0.0000   0.0000   0.0000
```

```
  0  7  7  0    4.0000  45.8264   0.9000  -4.0000   0.0000   0.0000   0.0000
  2  1  3  7   -1.5000  18.9285   0.3649  -6.1208   0.0000   0.0000   0.0000
  2  3  7  3    1.5000  -1.0000   0.2575  -6.2100   0.0000   0.0000   0.0000
  1  3  7  3   -1.4375  -0.8700   0.9861  -2.5424   0.0000   0.0000   0.0000
  7  3  7  3   -1.5000  21.5086  -1.0000  -4.8869   0.0000   0.0000   0.0000
  2  1  3  9    0.0000  84.3556   0.1000  -3.1953   0.0000   0.0000   0.0000
  1  1  3  9    0.0000  51.0461   0.1059  -7.2043   0.0000   0.0000   0.0000
  2  3  9  3   -0.2500   0.0100  -0.5000  -4.6984   0.0000   0.0000   0.0000
  1  1  1 11    0.5000   0.1000   0.4683 -11.5274  -1.7255   0.0000   0.0000
  2  1  1 11    0.0000  49.3871   0.2000 -10.5765  -1.7255   0.0000   0.0000
 11  1  1 11   -0.5000  95.4727  -0.2080  -4.8579  -1.7255   0.0000   0.0000
  0  1 11  0    4.0000  45.8264   0.9000  -4.0000   0.0000   0.0000   0.0000
  0 11 11  0    4.0000  45.8264   0.8897  -4.0000   0.0000   0.0000   0.0000
  2  3 13  3    0.0918  64.5743  -1.0000  -4.4228   0.0000   0.0000   0.0000
  1  1  1  1    0.0000   5.0000  -0.2000  -7.2626  -2.0000   0.0000   0.0000
  2  1  1  1    0.0000  46.5404  -0.0659  -8.0000  -2.0000   0.0000   0.0000
  1  1  1  1    0.0000  56.0716   0.1000  -4.5000  -2.0000   0.0000   0.0000
  0  1  1  0    0.0000  45.8264   0.3000  -4.0000   0.0000   0.0000   0.0000
  0  1  1  0    0.0000   1.0000   0.1000  -4.0000   0.0000   0.0000   0.0000
 13     ! Nr of hydrogen bonds;at1;at2;at3;Rhb;Dehb;vhb1
  3  2  3    2.1200  -3.5800   1.4500  19.5000
  3  2  4    1.6787  -3.9601   1.4500  19.5000
  4  2  3    1.5585  -3.9305   1.4500  19.5000
  4  2  4    1.9336  -5.8831   1.4500  19.5000
  3  2  5    1.5000  -2.0000   1.4500  19.5000
  4  2  5    1.5000  -2.0000   1.4500  19.5000
  5  2  3    1.5000  -2.0000   1.4500  19.5000
  5  2  4    1.5000  -2.0000   1.4500  19.5000
  5  2  5    1.5000  -2.0000   1.4500  19.5000
  3  2 11    1.5033  -0.0100   1.4500  19.5000
 11  2  3    1.7547  -0.2589   1.4500  19.5000
  3  2 10    1.8833  -3.6250   1.4500  19.5000
 10  2  3    1.8487  -0.0100   1.4500  19.5000
```
